# Studying light-harvesting models with superconducting circuits

**DOI:** 10.1038/s41467-018-03312-x

**Published:** 2018-03-02

**Authors:** Anton Potočnik, Arno Bargerbos, Florian A. Y. N. Schröder, Saeed A. Khan, Michele C. Collodo, Simone Gasparinetti, Yves Salathé, Celestino Creatore, Christopher Eichler, Hakan E. Türeci, Alex W. Chin, Andreas Wallraff

**Affiliations:** 1 Department of Physics, ETH Zurich, CH-8093 Zürich, Switzerland; 2 Cavendish Laboratory, University of Cambridge, J. J. Thomson Avenue, Cambridge, CB3 0HE UK; 3 Department of Electrical Engineering, Princeton University, Princeton, NJ 08544 USA

## Abstract

The process of photosynthesis, the main source of energy in the living world, converts sunlight into chemical energy. The high efficiency of this process is believed to be enabled by an interplay between the quantum nature of molecular structures in photosynthetic complexes and their interaction with the environment. Investigating these effects in biological samples is challenging due to their complex and disordered structure. Here we experimentally demonstrate a technique for studying photosynthetic models based on superconducting quantum circuits, which complements existing experimental, theoretical, and computational approaches. We demonstrate a high degree of freedom in design and experimental control of our approach based on a simplified three-site model of a pigment protein complex with realistic parameters scaled down in energy by a factor of 10^5^. We show that the excitation transport between quantum-coherent sites disordered in energy can be enabled through the interaction with environmental noise. We also show that the efficiency of the process is maximized for structured noise resembling intramolecular phononic environments found in photosynthetic complexes.

## Abstract (web summary)

Investigating photosynthesis processes in biological samples is challenging due to their complex and disordered structure. Based on analog quantum simulations with superconducting quantum circuits, the authors show how the interplay of quantum coherence and environmental interactions affects energy transport.

## Introduction

It is well accepted that the microscopic properties of all matter being composed of atoms and molecules are governed by the laws of quantum physics. At macroscopic scales, however, coherent quantum phenomena are frequently suppressed by the interaction with the environment. An intensely studied open question is, whether quantum mechanics plays an important functional role in biological processes. Examples of such processes are magnetoreception in birds, olfaction, and light harvesting, all studied in a field referred to as quantum biology^1–3^. In particular, quantum-coherent effects were observed in photosynthetic complexes by 2D electron spectroscopy at near-ambient conditions^4–6^, which stimulated both experimental and theoretical work on light harvesting.

In a photosynthetic process, light is captured in a molecular complex acting as an antenna. The created excitation is then relayed toward a reaction center through a network of chlorophyll molecules forming pigment protein complexes, such as the well studied Fenna–Matthews–Olson (FMO) complex^1,2^. At the reaction center the excitation enables the synthesis of energy-rich molecules, e.g., adenosine triphosphate, relevant for supplying chemical energy throughout an organism. An excitation of an individual chlorophyll molecule is carried by a single chromophore whose highest occupied and lowest unoccupied molecular orbital can be approximated as a two-level system. The chlorophyll molecules form the sites of a network through which the individual excitations are transported. The energy levels of the individual sites and the coupling between the sites are affected by both static and dynamic disorder, which in uniform systems suppresses energy transfer between sites.

High-efficiency energy transport between disordered sites is suggested to be enabled through the interaction of the individual sites with vibrational modes of the protein scaffold into which the chlorophylls are embedded. A number of theoretical models have been developed to put this mechanism of efficient energy transport onto a solid footing^7–11^. In particular, it has been suggested that interactions between the energy levels of the chlorophyll molecules and the highly structured phononic environment of the protein scaffold enhance directed excitation transport^12–15^.

The direct verification of these models is challenging due to the intricate structure and the limited control obtainable over photosynthetic complexes. Despite a number of theoretical studies, noise-assisted energy transport (NAT) in biological systems has so far only been phenomenologically investigated on simple model systems with limited control over its parameters. Energy transport between two molecules placed on a substrate was studied with scanning tunneling microscopy^16^, using classical optics disorder was shown to break destructive interference and increase optical transmission^17–19^; similarly, disorder in the coupling parameter was shown to increase energy flow using classical electronic circuits^20^ and in genetically engineered molecular systems energy transport was controlled by adjusting inter-chromophoric distances^21,22^. Recently, a programmable nanophotonic processor was used to study the transport properties in disordered systems^23^.

In this work we demonstrate the use of superconducting quantum circuits^24,25^ to test models describing important aspects of photosynthesis, such as photon absorption and noise assisted excitation transport, with unprecedented control in an engineered quantum system. We realize a small network of coherently coupled two-level systems with in situ tunable parameters interacting with an engineered environment, inspired by the proposal of Mostame et al.^26,27^. Superconducting circuits are particularly well suited for this task, since versatile devices can be realized with a high degree of accuracy, and can be controlled and probed experimentally using well-developed techniques^28^. Based on recent developments in circuit design, control, and measurement with efforts aimed at realizing circuits for quantum information processing^29–31^, it seems likely that our approach could be extended to study more complex quantum networks. We experimentally demonstrate energy transport assisted by structured and unstructured environmental noise for coherent and incoherent excitation and show that its efficiency can approach unity. We also observe static coherences, even under incoherent excitation, and demonstrate good understanding of the full system dynamics.

## Results

### Sample and spectroscopic characterization

**Fig. 1:**
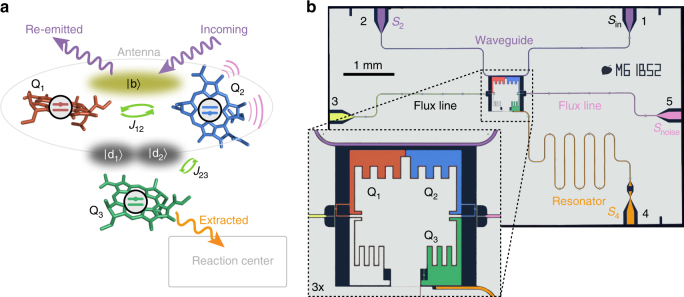
Model system for light harvesting and its superconducting circuit realization. **a** Schematic of three coupled chlorophyll molecules hosting chromophores modeled as three qubits Q_1,2,3_ (red, blue, and green). The strongly coupled Q_1_ and Q_2_ hybridize with coupling strength *J*_12_ into a bright state |b〉 (olive cloud) and dark states (dark gray clouds). Q_2_ is coupled to Q_3_ with a coupling strength *J*_23_ which forms the dark hybridized states |d_1_〉 and |d_2_〉. The incident, the re-emitted, and the harvested radiation are indicated by arrows. Pink lines indicate that Q_2_ is subjected to environmental noise. **b** False color micrograph of the superconducting circuit. Transmon qubits Q_1_ and Q_2_ (red, blue) are capacitively coupled to a transmission line (purple) and Q_3_ (green) is capacitively coupled to a high-emission-rate resonator (orange). Excitation radiation (*S*_in_) is applied through port 1 of the transmission line. The re-emitted (*S*_2_) and extracted radiation (*S*_4_) are detected at port 2 and 4, respectively. Low-frequency noise with an engineered spectral density, modeling the environment (*S*_noise_), is applied to Q_2_ via the flux line at port 5

We implement a simplified model of a pigment protein complex consisting of three coupled chlorophyll molecules, labeled Q_1,2,3_ in Fig. 1a. The corresponding Hamiltonian is described in Supplementary Note 1. This is the smallest system which incorporates all relevant elements, such as excitation trapping, energy mismatch, excitation delocalization, and dark and bright states, necessary for studying noise-assisted transport^1,9,11,13–15^. Although current technology allows building larger systems capable of investigating, for example the full FMO complex with eight sites, we are convinced that it is a necessary first step to explore this novel approach on a simple model system. We realize two-level systems with individually tunable transition frequencies as transmon qubits^32^ in a superconducting circuit (Fig. 1b). The dipole–dipole coupling between molecules Q_1_ and Q_2_ forms symmetric and antisymmetric, bright |b〉 and dark |d〉 state superpositions of the individual qubit excited states |q_1_〉 and |q_2_〉^33–35^. We realize the dipole–dipole interaction by direct capacitive coupling between qubits Q_1_ and Q_2_ (Fig. 1b). We excite the bright state |b〉 through an open waveguide to which the two transmon qubits are coupled with equal strength^35^ modeling the excitation of the antenna part of the photosynthetic complex with photons propagating in free space. A third molecule Q_3_, coupled to Q_2_, acts as a trap for the excitation which is subsequently extracted by transfer to the reaction center. In our circuit, this trapping site is realized as a third qubit which extracts the excitation from the system through its Purcell-like coupling to a transmission line resonator effectively acting as the reaction center. We model the interaction of molecule Q_2_ with the environmental vibrational modes of the protein scaffold as fluctuations of its transition frequency. These fluctuations are induced by local magnetic fields acting on Q_2_. While noise can be applied to all qubits, we chose to study local noise, such as the one induced by a local environment of a pigment protein. When applying noise to several sites, we could study the effects of correlations in the noise have on the process.

**Fig. 2:**
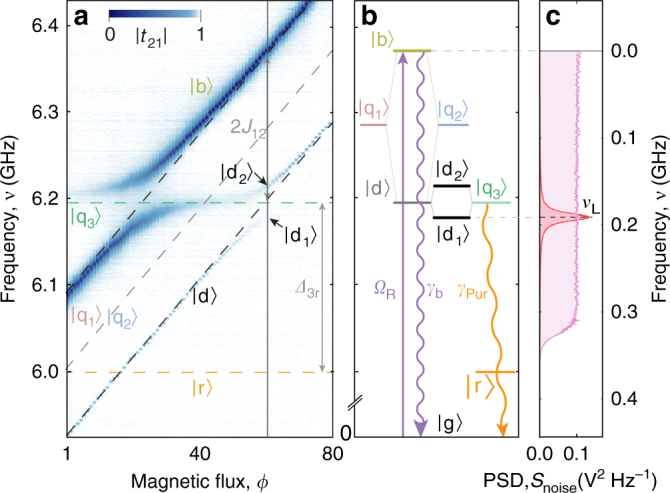
Measured spectrum, energy levels, and applied environmental noise spectra. **a** Transmission spectra ∣*t*_21_(*ω*)∣ of the three-qubit system measured through the transmission line as a function of magnetic flux. Here and in the following, spectral features are labeled by the target state (|q_1_〉, |q_2_〉, |q_3_〉, |b〉, |d〉, |d_1_〉, |d_2_〉, |r〉) reached in spectroscopic experiments from the ground state |g〉 of the system. **b** Energy-level diagram at the magnetic flux indicated by a gray vertical line in **a**. The resonant states |q_1_〉 and |q_2_〉 form bright |b〉 and dark |d〉 states. Furthermore, |q_3_〉 is resonant with the |d〉 state forming |d_1_〉 and |d_2_〉 doublet. Solid downward arrows indicate decay channels; the upward arrow indicates excitation via the waveguide. **c** Measured power spectral density (PSD) *S*_noise_ of the environmental low frequency noise applied to the flux line of Q_2_. White noise with 325 MHz cutoff is depicted in pink and Lorentzian noise with central frequency *ν*_L_ in red

We demonstrate a high degree of tunability of system parameters in a measurement of the frequency-dependent transmission coefficient |*t*_21_(*ω*)|, through the waveguide (Fig. 2a), keeping the transition frequency of Q_3_ fixed at *ω*_3_/2*π* = 6.198 GHz and linearly sweeping the transition frequencies of Q_1_ and Q_2_ maintaining *ω*_1_ = *ω*_2_. We tune the qubit transition frequencies by magnetic fields applied using a coil and two flux lines shorted close to the SQUID loops of transmon qubits Q_1_ and Q_2_ (see Supplementary Note 1). In this measurement, we observe that Q_1_ and Q_2_ form bright and dark states (|b〉, |d〉) with frequencies *ω*_b_ and *ω*_d_ separated by 2*J*_12_/2*π* = 173.4 MHz (see Supplementary Note 2). The bright state linewidth *γ*_b_/2*π* = 12.4 MHz is consistent with the sum of the individual qubit radiative linewidths *γ*_1_/2*π* = 7.39 MHz and *γ*_2_/2*π* = 6.57 MHz dominated by the coupling to the waveguide. This indicates superradiance of the coupled two-qubit system^35,36^. The subradiant dark state |d〉 has a narrow linewidth of *γ*_d_/2*π* = 0.29 MHz limited by the residual asymmetry in Q_1_ and Q_2_ parameters (see Supplementary Note 1) with a bright to dark state linewidth ratio of *γ*_b_/*γ*_d_ = 43. Dark states have been suggested to improve the efficiency of biologically inspired photocells by protecting the excitation from re-emission^14,37^. For *ω*_1_/2*π* = *ω*_2_/2*π* = 6.285 GHz (solid vertical line in Fig. 2a) the dark state |d〉 coherently hybridizes with |q_3_〉 forming a doublet |d_1_〉 and |d_2_〉 split by 2*J*_d3_/2*π* = 37 MHz consistent with the individual qubit couplings (see Fig. 2b and Supplementary Notes 1 and 2). We detune *ω*_3_ from the fixed frequency *λ*/2 resonator state |r〉 at *ω*_r_/2*π* = 6.00 GHz by Δ_3r_ = *ω*_3_ − *ω*_r_. This sets the radiative Purcell decay rate^38^ *γ*_Pur_/2*π* = 20MHz of Q_3_ (see Supplementary Note 3) effectively modeling the energy extraction rate at the reaction center. As desired, all relevant microwave frequency system parameters are consistently scaled by a factor of ~10^5^ relative to the optical-frequency energy scales of the FMO complex^26^.

### Excitation transfer with uniform white noise

**Fig. 3:**
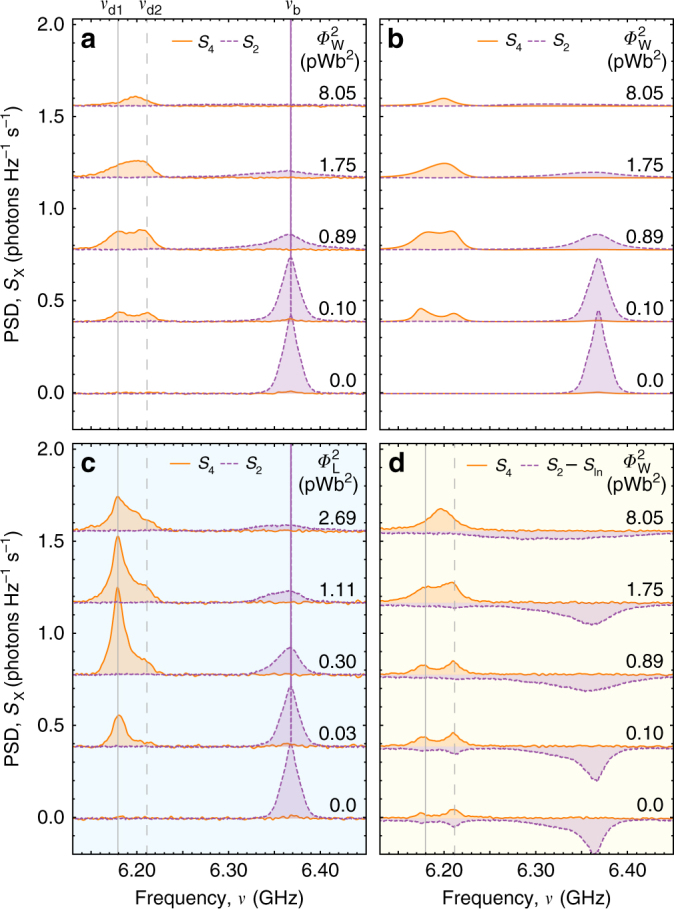
Measured power spectral densities (PSD). PSD of radiation extracted from the resonator *S*_4_(*ω*) (solid orange lines) and re-emitted into the transmission line *S*_2_(*ω*) (dashed purple lines) for coherent excitation as a function of **a** white noise power ${\Phi }_{\mathrm{W}}^2$ and **c** Lorentzian noise power ${{\Phi }}_{\mathrm{L}}^2$. **b** Master equation calculations of *S*_4_(*ω*) and *S*_2_(*ω*) for coherent excitation as a function of ${{\Phi }}_{\mathrm{W}}^2$. **d** Measured PSD for incoherent excitation as a function of ${{\Phi }}_{\mathrm{W}}^2$. Dashed purple lines represent a difference between measured *S*_2_(*ω*) and PSD of incoherent microwave radiation *S*_in_. PSD for different noise powers are displaced by 0.2 photons s^−1^ Hz^−1^

To study energy transfer through the circuit, we tune Q_1_ and Q_2_ into resonance to form bright and dark states at frequencies *ω*_b_/2*π* = *ν*_b_ = 6.371 GHz and *ω*_d_/2*π* = 6.198 GHz. Qubit Q_3_ is tuned into resonance with the dark state *ω*_3_ = *ω*_d_ creating two resonances at *ω*_d1_/2*π* = *ν*_d1_ = 6.179 GHz and *ω*_d2_/2*π* = *ν*_d2_ = 6.216 GHz. We coherently excite the bright state |b〉 through port 1 of the device with a continuous tone at frequency *ω*_b_ and amplitude corresponding to a bright state Rabi frequency of *Ω*_R_/2*π* = 14 MHz (see Supplementary Note 4). We measure the power spectral density (PSD) *S*_2_(*ω*) of the photons scattered along the waveguide into port 2 of the device characterizing the re-emission from the absorption site. *S*_2_(*ω*) displays a narrow coherent peak at *ω*_b_ due to elastically (Rayleigh) scattered photons and a broad resonance fluorescence spectrum with a width given by *γ*_b_ due to the inelastically scattered photons (bottom purple line in Fig. 3a). With increasing drive amplitude we observe a bright state Mollow triplet^35^, see Supplementary Fig. 4. Due to the energy mismatch of the bright state |b〉 and the dark state doublet (|d_1_〉, |d_2_〉) no excitations are transferred to qubit Q_3_ and thus no photons are detected at the resonator port 4, as shown by the vanishing PSD *S*_4_(*ω*) at ${{\Phi }}_{\mathrm{w}}^2 = 0$ pWb^2^ (bottom orange line in Fig. 3a). In our model system, no energy is transferred from the antenna to the reaction center in the absence of environmental noise.

To engineer a broad environmental noise spectrum, such as the one generated by the combination of background thermal noise and overlapping vibrational modes of the protein scaffold present in light-harvesting systems^26^, we apply white Gaussian noise to port 5 inducing frequency fluctuations in Q_2_. The broad Markovian noise has a PSD of adjustable amplitude, constant up to a cutoff frequency of 325 MHz, characterized by its integrated flux noise power ${{\Phi }}_{\mathrm{W}}^2$ at qubit Q_2_ (see Supplementary Note 5 and Fig. 2c). We note that applying synthesized noise to |q_2_〉 effectively creates a classical environment that can be described by the Haken–Strobl–Reineker model^39,40^ for white noise. Applying classical, as opposed to quantum noise, offers a unique possibility to engineer noise with controllable PSD capable of creating environments that approximate those of pigment protein complexes^26^ without increasing complexity of the device design.

For small applied noise powers, we observe energy transfer from the bright |b〉 to the dark state doublet |d_1_〉, |d_2_〉 indicated by two resonances at frequencies *ω*_d1_ and *ω*_d2_ in the detected PSD *S*_4_(*ω*) (orange line at ${{\Phi }}_{\mathrm{W}}^2 = 0.1\,{\mathrm{pWb}}^{\mathrm{2}}$ in Fig. 3a). The excitation transport is enabled by those frequency components of the noise spectrum that bridge the energy difference 2*J*_12_ ± *J*_d3_ between bright |b〉 and dark states |d_1_〉, |d_2_〉. We have verified this aspect by reducing the bandwidth of the noise to below that energy difference in which case no energy transfer is observed. The emission linewidths, i.e., the emission rates of |d_1_〉 and |d_2_〉 into the resonator are determined by the Purcell decay rate *γ*_Pur_ (see Supplementary Note 3). The well-resolved doublet in the detected spectrum *S*_4_(*ω*) indicates that static coherences of the underlying quantum network are observable in noise induced transport. Based on the observations of the doublet, we expect beatings with frequency 2*J*_d3_ to be observable in temporally resolved measurements of the power at the extraction site.

With increasing applied noise power ${{\Phi }}_{\mathrm{W}}^2$, the power spectrum *S*_2_(*ω*) of the resonance fluorescence of the bright state |b〉 broadens due to the pure dephasing induced by the noise. From this measurement we determine the bright state pure dephasing rate $\gamma _\phi ^{\mathrm{b}}$ in dependence on the applied white noise power ${{\Phi }}_{\mathrm{W}}^2$ (see Supplementary Note 6). The extracted power *S*_4_(*ω*) first increases with increasing noise power ${{\Phi }}_{\mathrm{W}}^2$ while the doublet remains resolved. At noise powers above ${{\Phi }}_{\mathrm{W}}^2 \approx 2$ pWb^2^, the observed doublet transforms into a single resonance marking a crossover from the strong-coupling regime $( {2J_{{\mathrm{d}}3} > rsim {\it{\gamma }}_{\phi} ^{\mathrm{b}}} )$ to the weak-coupling regime $( {2J_{{\mathrm{d3}}} \lesssim {\it{\gamma }}_\phi ^{\mathrm{b}}} ),$ where the remaining resonance stems from the incoherently excited |q_3_〉 state (see Supplementary Note 7). Beyond this threshold, the extracted power decreases. For this simple situation of only three sites and Markovian noise, all essential features of the experimentally observed power spectra are consistent with both Lindblad master equation and Bloch–Redfield calculations (Fig. 3b and Supplementary Notes 8, 9).

**Fig. 4:**
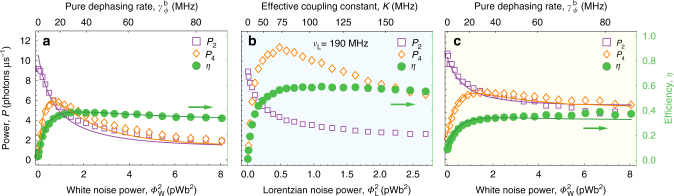
Total extracted power *P*_4_, re-emitted power *P*_2_, and transport efficiency *η* for **a** coherent excitation and white noise environments, **b** coherent excitation and Lorentzian noise environments, and **c** incoherent excitation and white noise environments. Data are plotted as a function of white noise power $\Phi _{\mathrm{W}}^2$ or equivalently bright state dephasing rate $\gamma _\phi ^{\mathrm{b}}$ for **a** and **c** and as a function of Lorentzian noise power $\Phi _{\mathrm{L}}^2$ or equivalently effective qubit-environment coupling constant *K* for **b**. Solid lines are results of master equation simulations (see Supplementary Note 9)

Integrating the measured PSD *S*_4_(*ω*) and *S*_2_(*ω*) while omitting contributions from elastic (Rayleigh) scattering, we find that the total power re-emitted from the bright state into the waveguide in forward direction *P*_2_ decreases monotonically as a function of applied noise power $\Phi _{\mathrm{W}}^2$ (open purple squares in Fig. 4a). In contrast, the total power detected at the extraction site *P*_4_ first increases rapidly with $\Phi _{\mathrm{W}}^2$, exhibits a pronounced maximum and then decreases again (open orange diamonds in Fig. 4a). The increase for small dephasing rates is a consequence of noise-induced incoherent transitions between bright and dark states^14,41^ allowing the system to overcome the energy mismatch.

From the integrated powers we calculate the transfer efficiency of the excitation from the absorption site to the extraction site as *η* = *P*_4_/(*P*_4_ + 2*P*_2_). The Factor 2 accounts for the bidirectional character of the bright state resonance fluorescence^35^, i.e., equal powers are emitted in forward and backward direction, while we detect only in forward direction. The transport efficiency *η* (green circles in Fig. 4a) shows a rapid increase from zero, a broad maximum of $\eta _{\mathrm{W}}^{{\mathrm{max}}} = 39\%$, and then a slow decrease with increasing pure dephasing rate $\gamma _\phi ^{\mathrm{b}}$ (top axis in Fig. 4a), which are the characteristic features of noise assisted transport^8–10^. The decrease in efficiency above an optimal noise power is due to dephasing-induced population localization, also referred to as the quantum Zeno effect^8–10^.

The measured integrated powers and hence the efficiency are in good agreement with results from master equation simulations (solid lines in Fig. 4a). Using rate equations (Supplementary Note 10), we show that the maximal efficiency is $\eta _{\mathrm{W}}^{{\mathrm{max}}} \approx (1 - \gamma _{\mathrm{b}}/\gamma _{{\mathrm{Pur}}})$ approaching 100% for *γ*_b_ ≪ *γ*_Pur_. Although small *γ*_b_ maximizes the transfer efficiency^10^, the total extracted power at optimal applied noise is proportional to *γ*_b_. Therefore for practical light harvesting applications one may choose to maximize output power while compromising on efficiency^14,41^. Similarly, we have not chosen a smaller *γ*_b_ in our experiment to maximize the efficiency, but opted for a larger extracted power to achieve a high signal-to-noise ratio at an acceptable integration time. Finally, we note that in our data the maximum efficiency $\eta _{\mathrm{W}}^{{\mathrm{max}}}$ occurs at the strong-to-weak coupling crossover $2J_{{\mathrm{d3}}} \approx \gamma _\phi ^{\mathrm{b}}$. At this point, the transfer rate $\gamma _\phi ^{\mathrm{b}}$ between $\left| {\mathrm{b}} \right\rangle$ and $\left| {\mathrm{d}} \right\rangle$ is comparable to the transfer rate between |d〉 and |q_3_〉, which is approximately given by $2J_{{\mathrm{d3}}}^2/\gamma _\phi ^{\mathrm{b}}$ (see Supplementary Note 10). This experimentally demonstrates the interplay between quantum coherent effects and classical dephasing enhancing excitation transport.

### Excitation transfer with lorentzian environment

It has been conjectured that structured environmental noise, such as the one originating from long-lived vibrational modes of chlorophyll molecules in photosynthetic complexes, can further enhance the energy transfer efficiency between the disordered molecular sites of the network in a scenario known as the phonon antenna mechanism^1,12^. To demonstrate this concept, we apply environmental noise with Lorentzian PSD, characterized by its central frequency *ν*_L_, its width Δ*ν*_L_ and amplitude (Fig. 2c), to qubit Q_2_. We select a fixed bandwidth Δ*ν*_L_ = 10 MHz, which in good approximation corresponds to the scaled linewidth of the environmental noise expected from vibrational modes in natural photosynthetic complexes^15,26^. Since Δ*ν*_L_ is comparable to the decay rates *γ*_b_ and *γ*_Pur_, the spectral properties of the noise effectively create a non-Markovian environment (see Supplementary Note 11). Initially, we choose the Lorentzian central frequency *ν*_L_ = 190 MHz to be resonant with the |b〉 to |d_1_〉 frequency difference *Δ*_b,d1_. The qualitative features of the measured PSD *S*_2_(*ω*) and *S*_4_(*ω*) and their dependence on the integrated applied noise power $\Phi _{\mathrm{L}}^2$ (Fig. 3c) are comparable to the white noise case (Fig. 3a) with some distinct differences. As a direct consequence of applying Lorentzian noise resonant at *Δ*_b,d1_, *S*_4_(*ω*) exhibits a strong resonance exactly at *ω*_d1_ and a weaker resonance at *ω*_d2_. The excitation transfer can be interpreted as a two-photon process^42^ absorbing one photon from the coherent excitation field at frequency *ω*_b_ and emitting one photon into the environmental noise field at frequency *Δ*_b,d1_ or *Δ*_b,d2_. This effectively creates a transition from the joint ground state through the bright state |b〉 into the dark states |d_1_〉 or |d_2_〉 from which energy is extracted.

When we sweep the Lorentzian noise center frequency over a broad range from *ν*_L_ = 0 to 300 MHz at weak noise power $\Phi _{\mathrm{L}}^2 = 0.016$ pWb^2^, we observe two well-resolved maxima in transferred power *P*_4_ when the noise is resonant with the bright to dark state frequency differences *Δ*_b,d1_ and *Δ*_b,d2_ (Supplementary Fig. 8). In contrast, no energy transfer is observed when the noise is far detuned. Both observations clearly demonstrate the strong sensitivity of the energy transfer on the spectral properties of the environmental noise, and indicate that the noise-assisted transport is enabled by a narrow part of the noise PSD that matches the energy gap otherwise blocking the energy transfer in the system. This observation is consistent with stochastically averaged master equation simulations (see Supplementary Fig. 9 and Supplementary Note 12). We note that despite applying classical noise the dynamics induced by Lorentzian environment cannot be simulated with the HSR approach or even its extension for colored noise^43,44^, due to its strong non-Markovian character. Therefore, a full quantum numerical simulation is required at the cost of significant computational effort.

For Lorentzian environmental noise, the integrated detected powers also display the characteristic properties of noise-assisted transport as a function of $\Phi _{\mathrm{L}}^2$ (Fig. 4b) as discussed before for white noise (Fig. 4a). However, we note that the extracted power *P*_4_ is almost twice as large at the same bright state excitation amplitude (Fig. 4a, b) leading to increased maximal efficiency $\eta _{\mathrm{L}}^{{\mathrm{max}}} = 58$%. This indicates that structured environmental noise matching internal energy differences of the quantum network indeed enhances the efficiency of the energy transfer. Estimating the effective qubit-environment coupling constant *K* (see Fig. 4b and Supplementary Note 13), we observe that the maximum in efficiency coincides with *K*/2*π* ≈ 100 MHz, which is comparable to the inter-qubit coupling constant *J*_12_. This demonstrates that strong coupling between qubit and phononic modes is required to achieve maximal energy transport. Such a situation has been suggested to enhance the transport in cyanobacterial light-harvesting proteins, allophycocyanin, and C-phycocyanin^45^.

We have also applied a coherent tone with controlled frequency *ν*_c_ and amplitude to qubit Q_2_ through the environmental channel (port 5) (see Supplementary Note 14), and observe even larger extracted powers at the same bright state |b〉 input field amplitude and near unit transfer efficiency (see Supplementary Fig. 10c). While this case does not occur in natural light harvesting since the environment cannot be fully coherent, it represents an interesting limiting case. In natural light-harvesting systems exposed to incoherent environments, the highest transfer efficiency is realized for structured environmental noise with a narrow spectral density peaked at the frequency of the internal energy mismatch between the sites of the network. Any excess spectral width of the environmental noise leads to additional dephasing (see Supplementary Note 11), which in turn reduces the absorption and energy transfer efficiency. These aspects are clearly demonstrated by the presented set of experiments comparing energy transfer with white and Lorentzian noise spectral density.

### Incoherent excitation

In the final set of experiments we excite the qubit system with incoherent microwave radiation to mimic excitation of biological pigment protein complexes with sunlight. We engineer 0.95 GHz broad incoherent microwave radiation centered at *ω*_B_ that spans over all qubit transition frequencies (see Supplementary Note 15). The incoherent microwave power integrated over the bright state spectrum was adjusted to be equal to the drive power used for the case of coherent excitation. In this experiment we study the transport of incoherently created excitations as a function of applied white noise power.

Since it is not possible to distinguish between the broad incoming incoherent and the re-emitted radiation at port 2, we plot in Fig. 3d, the difference between detected power spectrum *S*_2_ at port 2 and the separately measured incoherent radiation spectrum *S*_In_. The difference spectrum corresponds to the sum of the absorbed and the re-emitted spectrum $\tilde S_2$.

When increasing the applied white noise power, we observe that *S*_4_(*ω*) and [*S*_2_(*ω*) − *S*_In_(*ω*)] show general features (Fig. 3d) similar to the ones observed for coherent excitation. However, in case of incoherent excitation, a finite power (*P*_4_ = 0.9 photons μs^−1^) is extracted at port 4 even in the absence of applied environmental white noise $\left( {\Phi _{\mathrm{W}}^2 = 0} \right)$. The observed extracted power is a result of direct excitation of the dark state, due to its finite coupling to the open waveguide. When the dark state is not completely dark, simultaneous incoherent excitation of bright and dark states reduces coherence between Q_1_ and Q_2_ and therefore increases dephasing of the system. Existence of dark states in photosynthetic complexes can therefore help protect the system against dephasing induced by incoherent excitation. The observation of the |d_1_〉, |d_2_〉 doublet in *S*_2_(*ω*) (Fig. 3d) demonstrates that static coherences can be observed for incoherent excitation, i.e., even in the absence of coherent sources, as long as the coherent coupling between the sites is larger than the total dephasing.

The maximum of the measured efficiency $\eta _{{\mathrm{W,inc}}{\mathrm{.}}}^{{\mathrm{max}}} = 38\%$ is smaller, but comparable to the case of coherent excitation with the maximum shifted toward higher applied environmental white noise powers. Comparable efficiencies are consistent with rate equation descriptions, where the efficiency is independent of the spectral and coherence properties of the excitation. On the other hand, the extracted power *P*_4_ is by more than a factor 2 larger at the highest environmental noise power ($\Phi _{\mathrm{W}}^2 = 8.05$ pWb^2^) compared to the coherent excitation case. This indicates that absorption of the incoherent photons is not as strongly affected by environmental dephasing as for coherent excitation, due to a persistent overlap between broadened bright state spectrum and spectrum of the incoherent irradiation. Similar conclusion can be made for energy transport induced by Lorentzian noise for incoherent excitation (see Supplementary Note 15).

## Discussion

In a proof of concept experiment we studied models of photosynthetic processes using superconducting quantum circuits. With a system of three coupled qubits we demonstrated how the interplay of quantum coherence and environmental interactions affects energy transport in a system with excellent control achievable over all relevant parameters. We expect this approach to be extensible to study other relevant aspects of light harvesting, such as time-resolved dynamics of the coherent excitation transfer; the role of quantum environments realizable in electronic circuit models as low frequency quantum harmonic oscillators^14,26^; and scaling to systems with a larger number of coherent sites such as the FMO complex. Furthermore, we expect similar approaches to be applicable not only to study light-harvesting processes but also other interesting aspects of quantum biology such as the sense of smell in animals, humans, and magnetoreception in birds^1,2^. It could also be interesting to evaluate the potential of the techniques presented here to model processes in quantum chemistry and search for potential future applications of related methods to support, for example, the design of catalysts, e.g., for nitrogen fixation, or biomolecular compounds for drug development.

### Data availability

The authors declare that the data supporting the findings of this study are available within the article and its Supplementary Information files or from the corresponding authors on reasonable request.

## Electronic supplementary material

Supplementary Information

Peer Review File
