# Publisher Correction: Studying light-harvesting models with superconducting circuits

**DOI:** 10.1038/s41467-018-04655-1

**Published:** 2018-06-08

**Authors:** Anton Potočnik, Arno Bargerbos, Florian A. Y. N. Schröder, Saeed A. Khan, Michele C. Collodo, Simone Gasparinetti, Yves Salathé, Celestino Creatore, Christopher Eichler, Hakan E. Türeci, Alex W. Chin, Andreas Wallraff

**Affiliations:** 1 Department of Physics, ETH Zurich, CH-8093 Zürich, Switzerland; 2 Cavendish Laboratory, University of Cambridge, J. J. Thomson Avenue, Cambridge, CB3 0HE UK; 3 Department of Electrical Engineering, Princeton University, Princeton, NJ 08544 USA

Correction to: *Nature Communications* 10.1038/s41467-018-03312-x, published online 02 March 2018

The original HTML version of this Article contained an error in the fourth sentence of the fourth paragraph of the ‘Excitation transfer with uniform white noise’ section of the Results, which incorrectly read ‘At noise powers above $\mathit{\Phi} _{\mathrm{W}}^2 \approx 2{\mathrm{pWb}}^2,$ the observed doublet transforms into a single resonance marking a crossover from the strong-coupling regime $(2J_{{\mathrm{d}}3} > rsim\gamma _\varphi ^{\mathrm{b}})$ to the weak-coupling regime $(2J_{{\mathrm{d}}3} \lesssim \gamma_\varphi^{\mathrm{b}})$, where the remaining resonance stems from the incoherently excited $\left| {{\mathrm{q}}_3} \right.\rangle$ state (see Supplementary Note 7).’ The correct version states ‘$(2J_{{\mathrm{d}}3} \,\gtrsim\, \gamma_\varphi^{\mathrm{b}})$’ in place of ‘$2J_{{\mathrm{d}}3} \,rsim \,\gamma _\varphi ^{\mathrm{b}}$’. This has been corrected in the HTML version of the Article.

The original PDF version of this Article incorrectly stated that ‘Correspondence and requests for materials should be addressed to A. Pčn.’, instead of the correct ‘Correspondence and requests for materials should be addressed to A. Potočnik’. This has been corrected in the PDF version of the Article.

