# Supplementary material for: Studying light-harvesting models with superconducting circuits
Source: Nat Commun. 2018 Mar 2;9:904. doi: 10.1038/s41467-018-03312-x (PMC5834603; doi:10.1038/s41467-018-03312-x)
Supplement: Supplementary file 1 — Supplementary Information [file 41467_2018_3312_MOESM1_ESM.pdf]

Supplementary Information for the Article:

Studying Light-Harvesting Models with Superconducting Circuits

by A. Potočnik *et al.*

Supplementary Note 1: Sample and Experimental Setup

Three qubit sample is designed to simulate the following Hamiltonian in the rotating wave approximation:

$$\hat{\mathcal{H}}/\hbar = \sum_{j=1}^{3}\left[\frac{(\omega_j-\omega_{\rm in})}{2}\hat{\sigma}_j^z + \sum_{k<j} J_{kj}\left(\hat{\sigma}_k^+\hat{\sigma}_j^- + \hat{\sigma}_j^+\hat{\sigma}_k^-\right)\right] + (\omega_{\rm r}-\omega_{\rm in})\hat{a}^\dagger\hat{a} + g_3\left(\hat{a}^\dagger\hat{\sigma}_3^- + \hat{\sigma}_3^+\hat{a}\right) + \frac{\Omega_{\rm R1}}{2}(\hat{\sigma}_1^+ + \hat{\sigma}_1^-) + \frac{\Omega_{\rm R2}}{2}(\hat{\sigma}_2^+ + \hat{\sigma}_2^-). \tag{1}$$

Here $\hat{\sigma}_j^z$, $\hat{\sigma}_j^+ = (\hat{\sigma}_j^x + i\hat{\sigma}_j^y)/2$ and $\hat{\sigma}_j^- = (\hat{\sigma}_j^x - i\hat{\sigma}_j^y)/2$ are Pauli operators, $\hat{a}$ ($\hat{a}^\dagger$) is the annihilation (creation) operator of the resonator's harmonic mode with a transition frequency $\omega_{\rm r}$. ω_i are qubit transition frequencies and $\omega_{\rm in}$ is the input microwave frequency and also the frequency of the reference frame. J_{kj} are coupling coefficients between the qubits, g_3 is a coupling coefficient between Q_3 and resonator and $\Omega_{\rm R1}$ ($\Omega_{\rm R1}$) is the Rabi frequency for Q_1 (Q_2).

Transmon qubits are implemented with a grounded design, similar to X-mon qubits[1], to minimize the unwanted capacitive coupling between Q_1 and Q_3. Their arrangement (see Fig. 1b in the main text) yields capacitive coupling rates of $J_{12}/2\pi = 83.6$ MHz, $J_{23}/2\pi = 33.4$ MHz and an order of magnitude smaller $J_{13}/2\pi = 3.67$ MHz. All reported qubit parameters are determined at the qubit transition frequency of $\omega/2\pi = 6.28$ GHz. When extracting individual qubit parameters the other two qubits are detuned by at least 1.5 GHz. The measured maximum transition frequencies between the ground $|g\rangle$ and first excited state $|e\rangle$ are $\omega_1^{\rm max}/2\pi = 6.948$ GHz, $\omega_2^{\rm max}/2\pi = 6.694$ GHz and $\omega_3^{\rm max}/2\pi = 7.271$ GHz for the three qubits and their anharmonicities of the first-to-second excited state are $\alpha_1/2\pi = -140$ MHz, $\alpha_2/2\pi = -142$ MHz and $\alpha_3/2\pi = -137$ MHz. The spectroscopically measured pure dephasing rates of Q_1 and Q_2 are $\gamma_\phi^{(1)}/2\pi = 115$ kHz and $\gamma_\phi^{(2)}/2\pi = 82$ kHz. Q_1 and Q_2 are coupled to an open waveguide (transmission line) with coupling rates $\gamma_1/2\pi = 6.57$ MHz and $\gamma_2/2\pi = 7.39$ MHz. Q_3 is coupled to a $\lambda/2$ resonator with coupling coefficient $g/2\pi \approx 90$ MHz. The uncoupled resonator has a fundamental frequency of $\omega_{\rm r}/2\pi = 6.00$ GHz and a loaded quality factor of $Q_{\rm L} \approx Q_{\rm ext} \approx 55$ dominated by the external coupling. The transition frequencies of the three qubits are tuned by magnetic flux Φ_i,[2]

$$\omega_i(\Phi_i) \simeq (\omega_i^{\rm max} - \alpha_i)\sqrt{|\cos(\pi\Phi_i/\Phi_0)|} + \alpha_i, \tag{2}$$

where Φ_0 is the flux quantum, for $i = 1-3$ qubits. Magnetic flux is generated by applying DC currents to two flux lines (FL1, FL2) located close to the SQUID loops of Q_1 and Q_2 and to a superconducting coil coupled globally to all three qubits. Individual qubit frequency control is obtained by inverting the flux coupling matrix and applying appropriate currents.

Coherent microwave radiation (RF) generated at room temperature by a commercial source is thermalized and attenuated at the 4 K, 100 mK and 11 mK stages of dilution refrigerator and applied to the sample at port 1 of the waveguide (see schematic diagram in Supplementary Fig. 1). Radiation emitted from the waveguide and from the resonator is amplified with high-electron-mobility transistor (HEMT) amplifiers at 4 K followed by a chain of ultralow-noise (ULN) and low-noise (LN) amplifiers at room temperature. Three isolators are inserted between the sample and the HEMT amplifier to suppress the amplifier input noise propagating back to the sample. The radiation emitted from port 2 of the waveguide is filtered with a band-pass filter (BPF). The amplified signals are down-converted to an intermediate frequency (IF) of 250 MHz with an IQ mixer using a local oscillator (LO) tone and then digitized with an analog-to-digital converter (ADC). The digital signal in the measurement bandwidth of 250 MHz is then processed by a field-programmable-gate-array (FPGA) which determines the amplitude and the power spectral density of the signal[3]. Typically $2^{24} \approx 16\cdot 10^6$ samples are collected in about 15 min to obtain a single power spectral density $S(\omega)$ measurement. The low frequency noise is generated by an arbitrary waveform generator (AWG) and combined with the DC bias using a bias tee with a low-frequency cutoff of 5 kHz and then applied to FL2 (see Supplementary Fig. 1). Instead of an AWG we could use, for example, a heated resistor to generate low frequency white noise. However, AWG offers a unique advantage of *in-situ* control of both amplitude and shape of the noise power spectrum and has been used previously to create quasi thermal noise in circuit QED experiments[4].

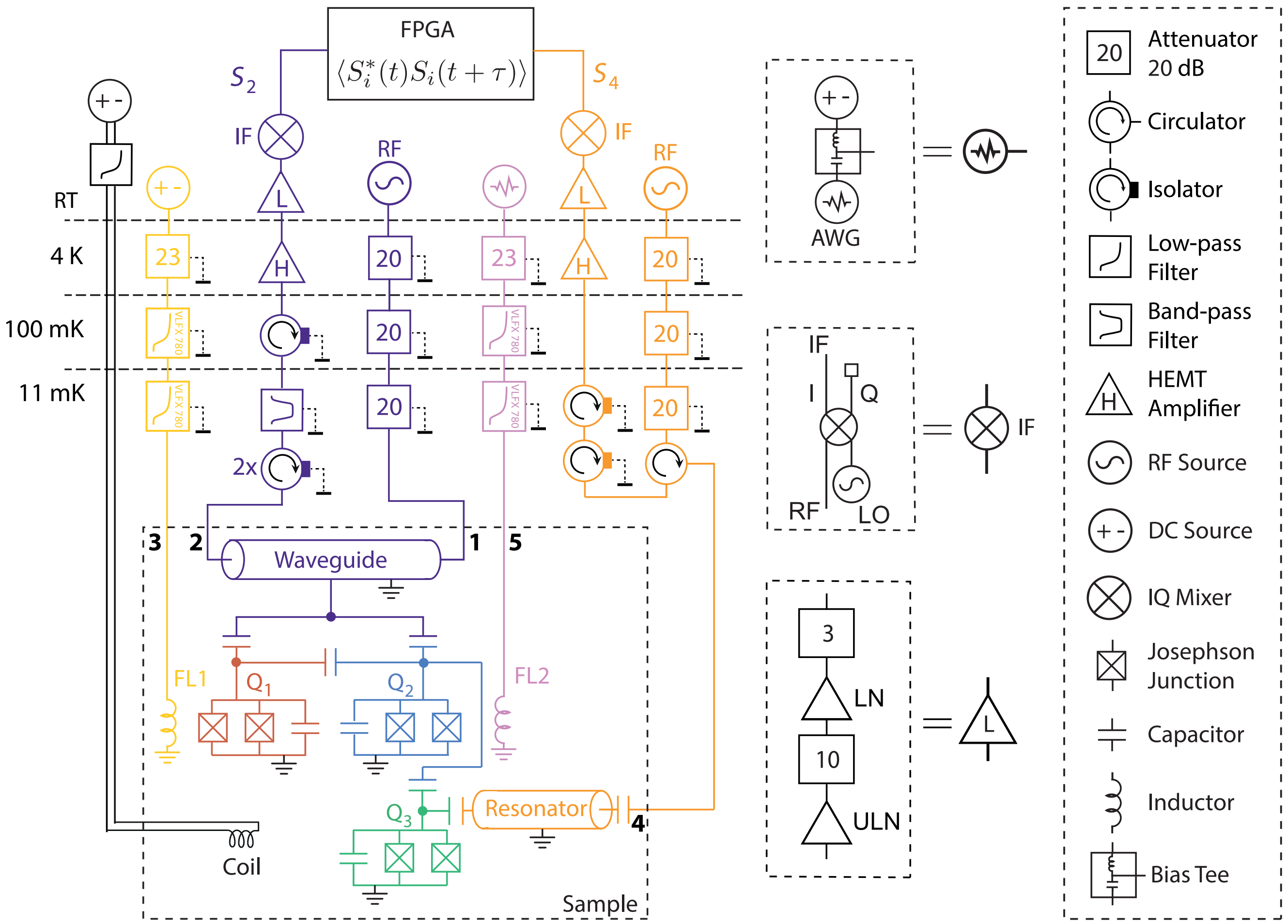

Supplementary Figure 1: Schematic of the experimental setup with complete wiring of electronic components inside and outside of the dilution refrigerator. Color code is the same as in Fig. 1b in the main text.

Supplementary Note 2: Description of Symmetric and Antisymmetric superposition of Q_1 and Q_2

On resonance, Q_1 and Q_2 excited states $|q_1\rangle$ and $|q_2\rangle$ form symmetric and antisymmetric (bright and dark) states $|b\rangle = (|q_1\rangle + |q_2\rangle)/\sqrt{2}$ and $|d\rangle = (|q_1\rangle - |q_2\rangle)/\sqrt{2}$. The formation of the hybridized states is observed as an avoided crossing in measurements of the transmission coefficient $|t_{21}|$ through the waveguide for magnetic flux chosen such that the bare frequencies of Q_1 and Q_2 cross, indicated by the black dashed lines in Supplementary Fig. 2a. Fully hybridized $|b\rangle$ and $|d\rangle$ states at zero detuning between Q_1 and Q_2 ($\Delta_{12} = 0$) are separated in energy by $2J_{12}$ (Supplementary Fig. 2b). We fit the measured transmission coefficient at the point of maximal hybridization using the expression[5]

$$t = 1 - \frac{\gamma_r}{\gamma_r + 2\gamma_\phi} \frac{1 - \frac{i\Delta}{\gamma_r/2+\gamma_\phi}}{1 + \left(\frac{\Delta}{\gamma_r/2+\gamma_\phi}\right)^2 + \frac{\Omega_R^2}{\gamma_r(\gamma_r/2+\gamma_\phi)}}, \quad (3)$$

with detuning $\Delta = \omega_i - \omega_{\mathrm{in}}$, excitation frequency ω_{in} and Rabi rate Ω_R. From the fit we extract the radiative decay rate γ_r, dominated by the coupling rate to the waveguide, and the pure dephasing rate γ_ϕ for both states. Here we neglect the non-radiative decay. We find a higher frequency bright state coupling rate of $\gamma_b/2\pi = 12.44$ MHz and pure dephasing rate of $\gamma_\phi^b/2\pi = 0.38$ MHz and a lower frequency subradiant state coupling rate of $\gamma_d/2\pi = 0.29$ MHz and comparable pure dephasing rate of $\gamma_\phi^d/2\pi = 0.55$ MHz.

Supplementary Note 3: Characterization of Q_3 and its Purcell Decay

We tune the Q_3 decay rate by adjusting the $|q_3\rangle$ transition frequency detuning from the extraction resonator frequency. When the resonator decay rate is large, the radiative decay rate of the qubit is enhanced by the Pur-

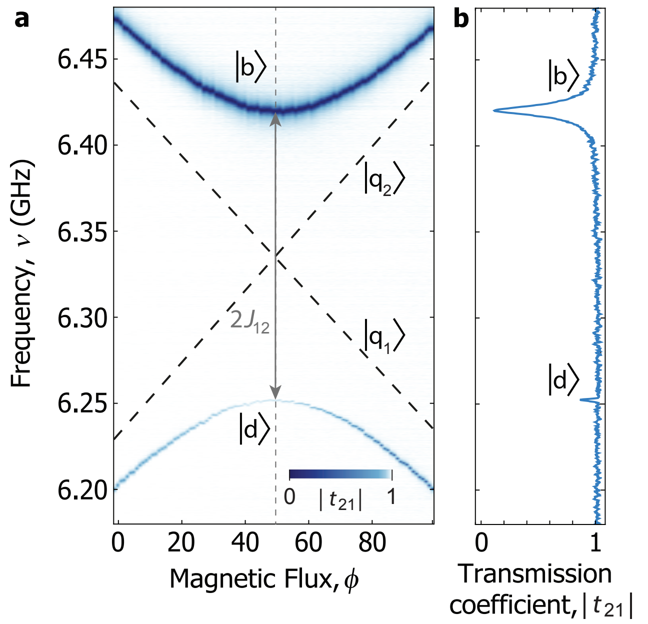

Supplementary Figure 2: Q_1, Q_2 avoided crossing. **a** Measured frequency dependent transmission coefficient $|t_{21}|$ of the waveguide as a function of magnetic flux. $|q_1\rangle$ and $|q_2\rangle$ transition frequencies linearly cross (black dashed lines). $|q_3\rangle$ is detuned by more than 1.5 GHz. **b** $|t_{21}|$ measurement as a function of frequency ν at $\phi = 49$ [vertical dashed line in **a**] where $|q_1\rangle$ and $|q_2\rangle$ are maximally hybridized.

cell effect[6,7]. The Purcell decay rate[8]

$$\gamma_{\rm Pur} = \frac{\kappa}{2} - \frac{\sqrt{2}}{2}\sqrt{-A + \sqrt{A^2 + (\kappa\Delta_{3\rm r})^2}}, \qquad (4)$$

with $A = \Delta_{3\rm r}^2 + 4g^2 - \kappa^2/4$, depends on the coupling g between the qubit and the resonator, the resonator decay rate κ and the detuning $\Delta_{3\rm r}$ between the qubit and the resonator. In the dispersive limit ($\Delta_{3\rm r} \gg g$), Eq. (4) reduces to the well known expression $\gamma_{\rm Pur} = \kappa\,(g/\Delta_{3\rm r})^2$[22,8]. The Purcell broadening of the Q_3 spectral line is observed in a reflection coefficient measurement $|t_{44}|$ at the resonator port 4 (Supplementary Fig. 3a). Fitting the spectral lineshape using master equation simulations we extract the Purcell decay rate $\gamma_{\rm Pur}$ which is a function of the tunable $|q_3\rangle$ frequency near the resonator fundamental mode $\omega_{\rm r}/2\pi = 6.00$ GHz (Supplementary Fig. 3b). The extracted values $\gamma_{\rm Pur}$ for positive detunings above the resonator frequency agree with Eq. (4) with coupling $g/2\pi = 90$ MHz and resonator decay rate $\kappa/2\pi = 110$ MHz. For $\omega_3/2\pi = 6.198$ GHz used in the presented experiments (indicated by the gray dashed line in Supplementary Fig. 3b) the Purcell decay rate is $\gamma_{\rm Pur}/2\pi \approx 20$ MHz.

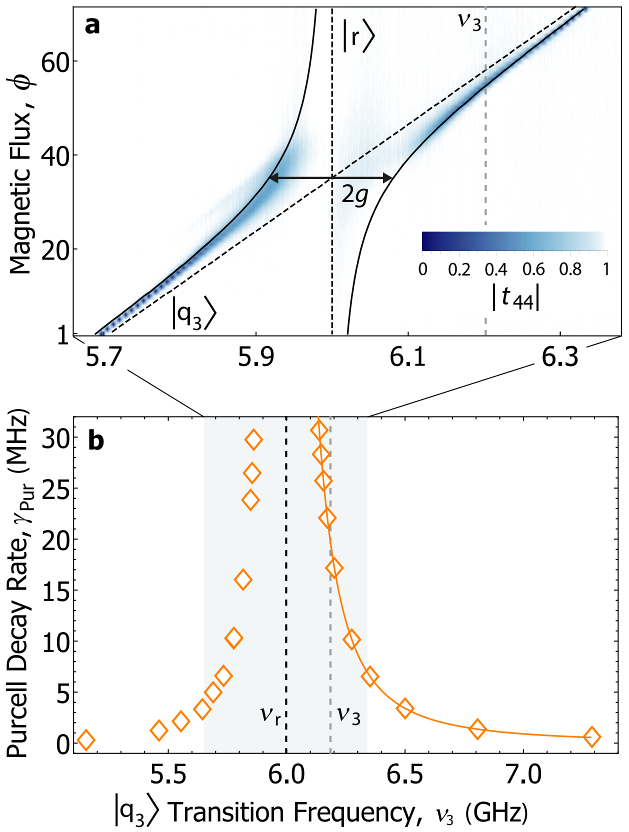

Supplementary Figure 3: Purcell decay of Q_3. **a** Frequency dependent reflection coefficient $|t_{44}|$ as a function of magnetic flux where the $|q_3\rangle$ transition frequency ω_3 is swept linearly and the resonator fundamental frequency $\omega_{\rm r}$ is fixed (displayed as black dashed lines). The gray dashed line corresponds to the $|q_3\rangle$ transition frequency $\omega_3/2\pi = 6.198$ GHz shown in Fig. 2b. **b** Q_3 Purcell decay rate $\gamma_{\rm Pur}$ as a function of Q_3 transition frequency extracted from the reflection coefficient measurements $|t_{44}|$. The resonator frequency $\omega_{\rm r}/2\pi = 6.00$ GHz is indicated with a black dashed line. The solid line is a fit to Eq. (4). The light blue area indicates the frequency range in **a** and the vertical gray dashed line indicates the $|q_3\rangle$ transition frequency, similar as in **a**.

Supplementary Note 4: Rabi Rate and PSD Calibration

The Rabi rate $\Omega_{\rm R}$ of the coherently driven bright $|b\rangle$ state was determined from measurements of bright state resonance fluorescence power spectra $S_2(\omega)$ (Supplementary Fig. 4). To obtain the Rabi rate $\Omega_{\rm R}/2\pi = 14$ MHz for the microwave powers used in our experiments we fit the resonance fluorescence spectrum to the Mollow triplet expression[9] assuming negligible pure dephasing and non-radiative decay. For larger applied microwave powers, the full Mollow triplet[10] of the bright state emerges with resolved side peaks. We determine the Rabi rate $\Omega_{\rm R}$ for these amplitudes from the frequency splitting between the central and the side peaks when fitting the spectra

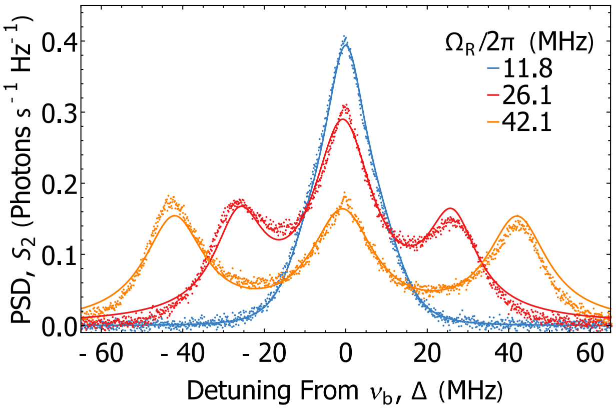

Supplementary Figure 4: Mollow triplet of the bright state. Measured power spectral density (PSD) $S_2(\omega)$ of the bright state resonance fluorescence emission spectrum at the output of the waveguide for indicated coherent microwave amplitudes given in terms of the extracted Rabi rate Ω_R. The microwave drive frequency was set to the bright state transition frequency ω_b. Solid lines are fits to the Mollow triplet spectrum[9] for $\Omega_R/2\pi = 11.8$ MHz and to the sum of three Lorentzian functions for $\Omega_R/2\pi = 26.1$ and 42.1 MHz.

with three Lorentzian lines. Fits to the data are shown with solid lines in Supplementary Fig. 4.

In order to calibrate the amplitude of the measured power spectral densities we performed the same power spectrum measurement on Q_2 with all other qubits detuned by more than 1.5 GHz. For strong coherent drive, when the qubit is saturated, and assuming that pure dephasing and non-radiative decay rates are negligible the integrated power of the measured Mollow triplet is equal to $\gamma_1/2$ photons per unit time. This allows us to express the magnitude of measured PSD in Photons s^{-1} Hz^{-1}.

Supplementary Note 5: Noise Generation

Low frequency noise is generated from a filtered random number time series consisting of $16 \cdot 10^6$ values. The bandwidth of the generated noise spans from 75 Hz to 600 MHz. The time series with a desired power spectral density $S(\omega)$ is constructed by first calculating the Gaussian random number sequence with a unit power spectral density $S(\omega) = 1$ and then applying a finite impulse response filter (FIR) with the frequency response function $H(\omega)$. We compensate AWG signal discritization distortions by pre-equalizing the digital noise series with an additional filter, constructed from the AWG output spectrum of an ideal white noise digital signal measured using a spectrum analyzer.

In this work we consider two distinct power spectral densities: (i) white noise with an exponential cutoff based on the Fermi-Dirac distribution

$$S_W(\omega) = \frac{A_W}{1 + e^{\frac{\omega-\omega_c}{\Delta\omega}}}, \tag{5}$$

where A_W is the amplitude of the function constant up to an exponential cutoff at $\omega_c/2\pi = 325$ MHz with a characteristic width $\Delta\omega/2\pi = 5.44$ MHz. (ii) Noise with a Lorentzian power spectral density:

$$S_L(\omega) = \frac{A_L}{1 + \left(\frac{\omega-\omega_L}{\Delta\omega_L/2}\right)^2}, \tag{6}$$

where A_L is the amplitude, $\omega_L/2\pi = 0 - 300$ MHz is the variable center frequency and $\Delta\omega_L/2\pi = 10$ MHz is the full width at half maximum. The noise power spectral densities in Fig. 2c in the main text are measured with a spectrum analyzer at the output of the AWG.

Supplementary Note 6: Pure Dephasing Rate Calibration

White noise applied to Q_2 increases the pure dephasing rate of $|q_2\rangle$ and consequently that of bright $|b\rangle$ and dark $|d\rangle$ states. The bright state pure dephasing rate γ_ϕ^b is determined from the resonance fluorescence power spectrum of the bright mode measured through the waveguide $S_2(\omega)$ for indicated applied noise powers Φ_W^2 (see Supplementary Fig. 5a). Measured spectra are fitted to the Mollow triplet expression[9] with the pure dephasing rate γ_ϕ^b as a free parameter and fixed center frequency ω_0, Rabi rate Ω_R and decay rate γ_b. The extracted pure dephasing rate γ_ϕ^b (see Supplementary Fig. 5b) shows an initial linear increase with applied white noise power Φ_W^2 as expected for ideal Markovian white power spectral density PSD[11]. Deviations from the linear dependence at higher noise powers originate from the finite cutoff of the engineered white noise (see Fig. 2c in the main text). This is corroborated by an excellent agreement between data and numerically calculated pure dephasing rate[11] using noise power spectral density with a finite cutoff frequency (solid line in Supplementary Fig. 5b).

Supplementary Note 7: White Noise PSD Analysis

We analyze power spectral densities $S_4(\omega)$ measured at the resonator as a function of applied environmental white noise power (see Fig. 3a and Supplementary Fig. 6a) by fitting the spectra with a sum of two Lorentzian functions

$$F(\nu) = \frac{a_1}{1 + 4\left(\frac{\nu-\nu_{01}}{\Delta\nu_1}\right)^2} + \frac{a_2}{1 + 4\left(\frac{\nu-\nu_{20}}{\Delta\nu_2}\right)^2}. \tag{7}$$

Here a_i is the amplitude, ν_{0i} the center frequency and $\Delta\nu_i$ the full width at half maximum of the i-th Lorentzian.

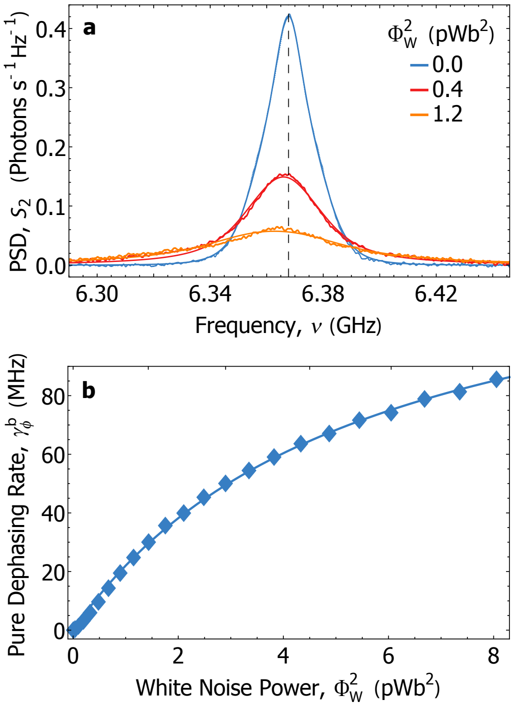

Supplementary Figure 5: Bright state dephasing rate. **a** Measured power spectral density $S_2(\omega)$ of the bright mode at port 2 of the waveguide for indicated environmental applied white noise powers Φ_W^2. Solid lines are fits to the Mollow-triplet spectrum[9], see text. **b** Pure dephasing rate γ_ϕ^b of the bright mode as a function of white noise power Φ_W^2 obtained from the fitted bright state resonance fluorescence spectra. The solid curve is a numerical calculation of the pure dephasing rate γ_ϕ^b for the white noise power spectral density with a finite frequency cutoff at 325 MHz (see Fig. 2c in the main text).

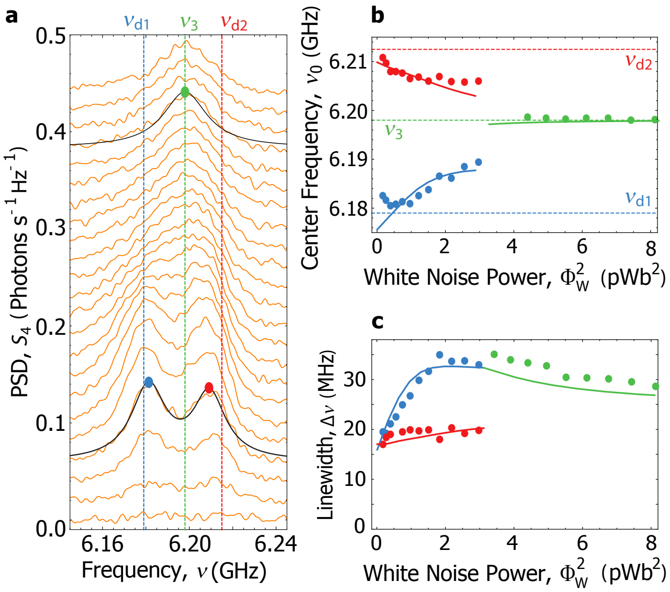

Supplementary Figure 6: Spectra of $|d_1\rangle$ and $|d_2\rangle$ dark states. **a** Power spectral density $S_4(\omega)$ at the resonator output port 4 for applied environmental white noise powers Φ_W^2 ranging from 0 to 8.1 pWb^2. Black solid curves show two selected fits to the data using two Lorentzian functions ($\Phi_W^2 = 0.6$ pWb^2) and a single Lorentzian function ($\Phi_W^2 = 6.1$ pWb^2). Vertical dashed lines indicate spectroscopically determined transition frequencies of $|d_1\rangle$ ($\nu_{d1} = 6.179$ GHz), $|d_2\rangle$ ($\nu_{d2} = 6.216$ GHz) and $|q_3\rangle$ ($\nu_3 = 6.198$ GHz) states. **b** Lorentzian center frequencies as a function of white noise power Φ_W^2 from the fits (red, blue and green dots). Horizontal dashed lines mark $|d_1\rangle$, $|d_2\rangle$ and $|q_3\rangle$ transition frequencies as in **a**. **c** Lorentzian full width at half maximum (fwhm) as a function of white noise power Φ_W^2 determined from fitting the power spectral densities indicated in **a**. Solid lines in **b** and **c** are results from master equation simulations.

For higher noise powers, for which the two peaks are not resolved anymore, we fit the data to a single Lorentzian. The two resonances centered near the spectroscopically determined transition frequencies ν_{d1} and ν_{d1} for low white noise powers gradually shift towards the bare $|q_3\rangle$ transition frequency ν_3 (Supplementary Fig. 6b). At the same time the linewidth of the lower frequency resonance corresponding to $|d_1\rangle$ significantly broadens while the one corresponding to $|d_2\rangle$ remains unchanged (see Supplementary Fig. 6c). The different dependence of the $|d_1\rangle$ and $|d_2\rangle$ resonances on the applied noise is attributed to an imperfect hybridization of the $|d\rangle$ and $|q_3\rangle$ states. From the analyzed data we determine the crossover from the strong to the weak coupling regime to occur at $\Phi_W^2 = 3.0 \pm 1.0$ pWb^2 corresponding to $\gamma_\phi^b/2\pi = 50 \pm 10$ MHz. This value is comparable to the $2J_{d3}/2\pi = 37$ MHz, which is in agreement with the crossover from strong to weak coupling as discussed in the main text.

Supplementary Note 8: Master Equation

Unitary dynamics of three coupled qubits where Q_3 is coupled to an extraction resonator and Q_1 and Q_2 are driven by a coherent tone applied to a waveguide is described by the Hamiltonian given in Eq. 1. In the case of ideal hybridization between Q_1 and Q_2 ($\omega_1 = \omega_2$) the Hamiltonian can be written in the bright and dark state bases as

$$\begin{aligned}\hat{\mathcal{H}}/\hbar = &\sum_{j=\mathrm{b,d,3}} (\omega_j - \omega_{\mathrm{in}})\hat{\sigma}_j^+\hat{\sigma}_j^- + (\omega_\mathrm{r} - \omega_{\mathrm{in}})\hat{a}^\dagger\hat{a} \\ &+ J_{\mathrm{b}3}\left(\hat{\sigma}_\mathrm{b}^+\hat{\sigma}_3^- + \hat{\sigma}_3^+\hat{\sigma}_\mathrm{b}^-\right) - J_{\mathrm{d}3}\left(\hat{\sigma}_\mathrm{d}^+\hat{\sigma}_3^- + \hat{\sigma}_3^+\hat{\sigma}_\mathrm{d}^-\right) \\ &+ g\left(\hat{a}^\dagger\hat{\sigma}_3^- + \hat{\sigma}_3^+\hat{a}\right) + \frac{\Omega_\mathrm{R}}{2}(\hat{\sigma}_\mathrm{b}^+ + \hat{\sigma}_\mathrm{b}^-),\end{aligned} \quad (8)$$

where $\hat{\sigma}_\mathrm{b}^\pm = (\hat{\sigma}_1^\pm + \hat{\sigma}_2^\pm)/\sqrt{2}$ and $\hat{\sigma}_\mathrm{d}^\pm = (\hat{\sigma}_1^\pm - \hat{\sigma}_2^\pm)/\sqrt{2}$ are bright and dark state creation and annihilation operators, $\omega_\mathrm{b} = \omega_1 + J_{12}$, $\omega_\mathrm{d} = \omega_1 - J_{12}$ are bright and

dark state transition frequencies, $J_{b3} = (J_{23} + J_{13})/\sqrt{2}$ and $J_{d3} = (J_{23} - J_{13})/\sqrt{2}$ are coupling rates between Q_3 and bright, and Q_3 and dark state, respectively, and $\Omega_R = \sqrt{2}\Omega_{R1}$ is the bright state Rabi frequency.

The full dynamics including the non-unitary terms is given by the Lindblad equation

$$\dot{\rho} = \mathcal{L}(\rho), \tag{9}$$

where ρ is the density matrix and $\mathcal{L}(\rho)$ is given by

$$\begin{aligned}\mathcal{L}(\rho) = &-\frac{i}{\hbar}\left[\hat{\mathcal{H}}, \rho\right] \\ &+ \gamma_b(1+n_{th})L(\sigma_b^-)\rho + \gamma_b n_{th} L(\sigma_b^+)\rho \\ &+ \gamma_d(1+n_{th})L(\sigma_d^-)\rho + \gamma_d n_{th} L(\sigma_d^+)\rho \\ &+ \frac{\gamma_\phi}{2} L(\sigma_2^+\sigma_2^- - \sigma_2^-\sigma_2^+)\rho \\ &+ \kappa L(a)\rho.\end{aligned} \tag{10}$$

Here $L(\sigma)$ is the Lindblad superoperator

$$L(\sigma)\rho = \hat{\sigma}\rho\hat{\sigma}^\dagger - \frac{1}{2}\left(\hat{\sigma}^\dagger\hat{\sigma}\rho + \rho\hat{\sigma}^\dagger\hat{\sigma}\right) \tag{11}$$

and $n_{th} < 0.01$, a typical thermal occupation for our experiments.

Supplementary Note 9: Numerical Simulations of Master Equation

Simulations of power spectral densities $S_2(\omega)$ at the waveguide output port 2 and the resonator $S_4(\omega)$ at port 4 as well as the integrated power at the waveguide P_2, the resonator P_4 and the transfer efficiency η are performed with QuTiP 3.1.0[12]. All simulations are done using the Lindblad master equation, which is sufficient for time independent decay channels, as well as the Bloch-Redfield master equation to account for the finite noise frequency cutoff (see Fig. 2c). Both methods yield identical results confirming that the noise PSD cutoff frequency ($\approx$325 MHz) is high enough for the interaction between our circuit and the environment to be considered in the Markovian approximation.

Waveguide and resonator spectra are calculated for the steady state $\dot{\rho} = 0$ via two-time correlation functions

$$S(\omega) = \int_{-\infty}^{\infty} \langle A(\tau)B(0)\rangle\, e^{-i\omega\tau}\, d\tau. \tag{12}$$

As a proxy for the waveguide emission we use the bright state $|b\rangle$ correlation function $\langle\hat{\sigma}_b^+(\tau)\hat{\sigma}_b^-(0)\rangle$ and for the resonator emission we use $\langle\hat{a}^\dagger(\tau)\hat{a}(0)\rangle$. The correct magnitude is achieved by multiplication with the respective radiative decay rates $\gamma_b/2$ and κ, where the factor 1/2 for the γ_b reflects the detection of only half of the photons emitted into the waveguide when measured only at port 2 and omitting port 1

$$S_2(\omega) \approx \frac{\gamma_b}{2}\int_{-\infty}^{\infty} \langle\hat{\sigma}_b^+(\tau)\hat{\sigma}_b^-(0)\rangle\, e^{-i\omega\tau}\, d\tau, \tag{13}$$

$$S_4(\omega) = \kappa\int_{-\infty}^{\infty} \langle\hat{a}^\dagger(\tau)\hat{a}(0)\rangle\, e^{-i\omega\tau}\, d\tau. \tag{14}$$

Description	Parameter	Value
$\lvert q_1\rangle$ transition frequency	$\omega_1/2\pi$	6.277 GHz
$\lvert q_2\rangle$ transition frequency	$\omega_2/2\pi$	6.277 GHz
$\lvert q_3\rangle$ transition frequency	$\omega_3/2\pi$	6.161 GHz
Resonator frequency	$\omega_r/2\pi$	6.000 GHz
Coupling between Q_1 and Q_2	$J_{12}/2\pi$	83.5 MHz
Coupling between Q_2 and Q_3	$J_{23}/2\pi$	33.4 MHz
Coupling between Q_1 and Q_3	$J_{13}/2\pi$	3.67 MHz
Coupling between Q_3 and the resonator	$g_3/2\pi$	90 MHz
$\lvert b\rangle$ state decay rate	$\gamma_b/2\pi$	12.4 MHz
Resonator decay rate	$\kappa/2\pi$	110 MHz
Bright state Rabi rate	$\Omega_R/2\pi$	14.0 MHz
Input field frequency	$\omega_{in}/2\pi$	6.368 GHz

Supplementary Table 1: System parameters used for simulations. All parameters are experimentally determined from spectroscopic measurements except g and κ which were adjusted within their experimental uncertainty.

As in the experimental analysis we obtain the full power as an integral of the power spectral density over frequency.

For the simulations shown in Fig. 3b and Figs. 4a,c, in the main text we use system parameters specified in Supplementary Table 1. In Figs. 4a,c of the main text the data is plotted against the bright state pure dephasing rate, which is related to the Q_2 pure dephasing rate as $\gamma_\phi^b = \gamma_\phi/2$ assuming that the bright state is an equal superposition of $|q_1\rangle$ and $|q_2\rangle$.

To reproduce experimental results for incoherent excitation a Lindblad master equation was solved without the last two terms in Eq. (1). A thermal occupation of $n_{th} = 0.3$ was used to compute the integrated re-emitted P_2 and extracted P_4 powers as well as the transport efficiency η shown with solid lines in Fig. 4c.

Supplementary Note 10: Rate Equations

The rate equations for the populations of the bright $|b\rangle$ state $p_b = \rho_{bb}$, dark $|d\rangle$ state $p_d = \rho_{dd}$ and $|q_3\rangle$ state $p_3 = \rho_{33}$ are derived from the Lindblad equation of motion [Eq. (9)] where the coupling of Q_3 to the resonator is approximated by an effective Purcell decay. The incoherent dephasing $\gamma_\phi/2\, L[\sigma_2^z]\rho$, with $\gamma_\phi = 2\gamma_\phi^b$ leads to a decay of all coherences involving Q_2

$$\dot{\rho}_{i2} = -\gamma_\phi\rho_{i2} \quad \forall i \neq 2. \tag{15}$$

In the bright/dark state basis this operator describes incoherent transport $\gamma_\phi^{\rm b}(L[\sigma_{\rm b}^+\sigma_{\rm d}^-] + L[\sigma_{\rm d}^+\sigma_{\rm b}^-])\rho$ between $|{\rm d}\rangle$ and $|{\rm b}\rangle$ towards an equilibrium population determined by

$$\frac{d}{dt}(\rho_{\rm bb} - \rho_{\rm dd}) = -2\underbrace{\gamma_\phi^{\rm b}}_{k_{\rm bd}}(\rho_{\rm bb} - \rho_{\rm dd}), \tag{16}$$

The bright state population $\dot\rho_{\rm bb} \propto \Omega_{\rm R}{\rm Im}(\rho_{\rm gb})$ is reduced by incoherent dephasing since the coherence between the ground and the bright state $\rho_{\rm gb}$ evolves as

$$\dot\rho_{\rm gb} \propto i\frac{\Omega_{\rm R}}{2}(\rho_{\rm gg} - \rho_{\rm bb}) - \frac{\gamma_{\rm b} + 2\gamma_\phi^{\rm b}}{2}\rho_{\rm gb}. \tag{17}$$

Thus absorption is reduced by dephasing when $\gamma_\phi^{\rm b} \gtrsim \Omega_{\rm R}$. In the steady state the bright state population can be written as

$$\dot\rho_{\rm bb} \propto \frac{\Omega_{\rm R}^2}{\gamma_{\rm b} + 2\gamma_\phi^{\rm b}}(\rho_{\rm gg} - \rho_{\rm bb}). \tag{18}$$

The coherent population transfer from $|{\rm d}\rangle$ to $|{\rm q}_3\rangle$ is defined by their coherence

$$\frac{d}{dt}(\rho_{\rm dd} - \rho_{33}) \propto -4J_{\rm d3}{\rm Im}(\rho_{\rm d3}), \tag{19}$$

which in turn is controlled by the coherent coupling and the incoherent dephasing

$$\dot\rho_{\rm d3} \propto -iJ_{\rm d3}(\rho_{33} - \rho_{\rm dd} + \rho_{\rm db}) - \frac{\gamma_{\rm Pur} + 2\gamma_\phi^{\rm b}}{2}\rho_{\rm d3}. \tag{20}$$

The scale at which coherent transport is expected to be reduced due to noise is $\gamma_\phi^{\rm b} \gtrsim J_{\rm d3}$. To derive the Förster transport rates $k_{\rm gb}$ between the ground $|{\rm g}\rangle$ and the bright state $|{\rm b}\rangle$ and the dark $|{\rm d}\rangle$ and $|{\rm q}_3\rangle$ state $k_{\rm d3}$ in the strong dephasing limit we assume that coherences are small, if they are not participating in transport, and that derivatives of coherences are negligible[13]. For $\rho_{db} \approx 0$ and $\dot\rho_{d3} = \dot\rho_{gb} = 0$ we therefore have

$$\rho_{\rm d3} = i\frac{-J_{\rm d3}}{\gamma_{\rm Pur}/2 + \gamma_\phi^{\rm b}}(\rho_{33} - \rho_{\rm dd}), \tag{21}$$

$$\rho_{\rm gb} = i\frac{\Omega_{\rm R}}{\gamma_{\rm b} + 2\gamma_\phi^{\rm b}}(\rho_{\rm gg} - \rho_{\rm bb}). \tag{22}$$

With the help of the above expressions we find the rate equations

$$\dot p_{\rm g} = -k_{\rm gb}(p_{\rm g} - p_{\rm b}) + \gamma_{\rm b}p_{\rm b} + \gamma_{\rm Pur}p_3, \tag{23}$$

$$\dot p_{\rm b} = k_{\rm gb}(p_{\rm g} - p_{\rm b}) - k_{\rm bd}(p_{\rm b} - p_{\rm d}) - \gamma_{\rm b}p_{\rm b}, \tag{24}$$

$$\dot p_{\rm d} = k_{\rm bd}(p_{\rm b} - p_{\rm d}) - k_{\rm d3}(p_{\rm d} - p_3), \tag{25}$$

$$\dot p_3 = k_{\rm d3}(p_{\rm d} - p_3) - \gamma_{\rm Pur}p_3, \tag{26}$$

for $|{\rm g}\rangle$, $|{\rm b}\rangle$, $|{\rm d}\rangle$ and $|{\rm q}_3\rangle$ populations with transfer rates

$$k_{\rm gb} = \frac{\Omega_{\rm R}^2}{\gamma_{\rm b} + 2\gamma_\phi^{\rm b}}, \tag{27}$$

$$k_{\rm d3} = \frac{4J_{\rm d3}^2}{\gamma_{\rm Pur} + 2\gamma_\phi^{\rm b}}, \tag{28}$$

$$k_{\rm bd} = \gamma_\phi^{\rm b}, \tag{29}$$

where state populations are bound by $p_i \in [0,\, 1]$. Since $\gamma_{\rm b} \approx \gamma_{\rm Pur}$ and $\Omega_{\rm R} \ll 2J_{\rm d3}$, the reduction of absorption for increasing $\gamma_\phi^{\rm b}$ happens before the complete decoupling of $|{\rm d}\rangle$ and $|{\rm q}_3\rangle$. From Eqs. (21) and (22) we see that population transfer between $|{\rm d}\rangle$ and $|{\rm q}_3\rangle$ is always coherent although $|{\rm d}\rangle$ is populated incoherently and the transfer is suppressed by the dephasing rate $\gamma_\phi^{\rm b}$.

Transfer efficiency is calculated using steady state solutions of Eqs. (23)-(26) as

$$\eta = \frac{\gamma_{\rm Pur}p_3}{\gamma_{\rm Pur}p_3 + \gamma_{\rm b}p_{\rm b}}, \tag{30}$$

where we assumed $p_g = 1$. The efficiency has a maximum at $\gamma_\phi^{\rm b} = \sqrt{2}J_{\rm d3} \approx J_{23}$ where it can be expressed as

$$\eta = \frac{1}{1 + \sqrt{2}\frac{\gamma_{\rm b}}{J_{\rm d3}} + \frac{\gamma_{\rm b}}{\gamma_{\rm Pur}} + \frac{\gamma_{\rm b}\gamma_{\rm Pur}}{4J_{\rm d3}^2}}. \tag{31}$$

Assuming that $2J_{\rm d3} \gg \gamma_{\rm b}, \gamma_{\rm Pur}$, the efficiency can be written as

$$\eta \approx (1 - \gamma_{\rm b}/\gamma_{\rm Pur}), \tag{32}$$

as stated in the main text.

Supplementary Note 11: Dephasing Rate due to Lorentzian Noise

Flux noise acting on Q_2 leads to an effective dephasing of the qubit with a dephasing rate depending on the noise power spectrum. In this section, we consider the effect of Lorentzian flux noise, in particular how it differs from white flux noise, and the emergence of non-Markovian effects. We study the essential physics with a single qubit model for Q_2 alone, described by the Hamiltonian

$$\mathcal{H}_2 = \frac{\omega_2}{2}\sigma_2^z + \xi(t)\sigma_2^z. \tag{33}$$

Here, ω_2 is the qubit transition frequency and $\xi(t)$ represents the input flux noise with power spectral density $S_{\xi\xi}(\omega)$,

$$\langle\xi(t)\rangle = 0\ ,\ S_{\xi\xi}(\omega) = \int_{-\infty}^{\infty} d\tau\ e^{-i\omega\tau}\langle\xi(\tau)\xi(0)\rangle \tag{34}$$

In the experiment, $S_{\xi\xi}(\omega)$ is either a flat spectrum up to a certain cutoff frequency $S_{\rm W}(\omega)$ [Eq. (5)] or a Lorentzian spectrum $S_{\rm L}(\omega)$ [Eq. (6)].

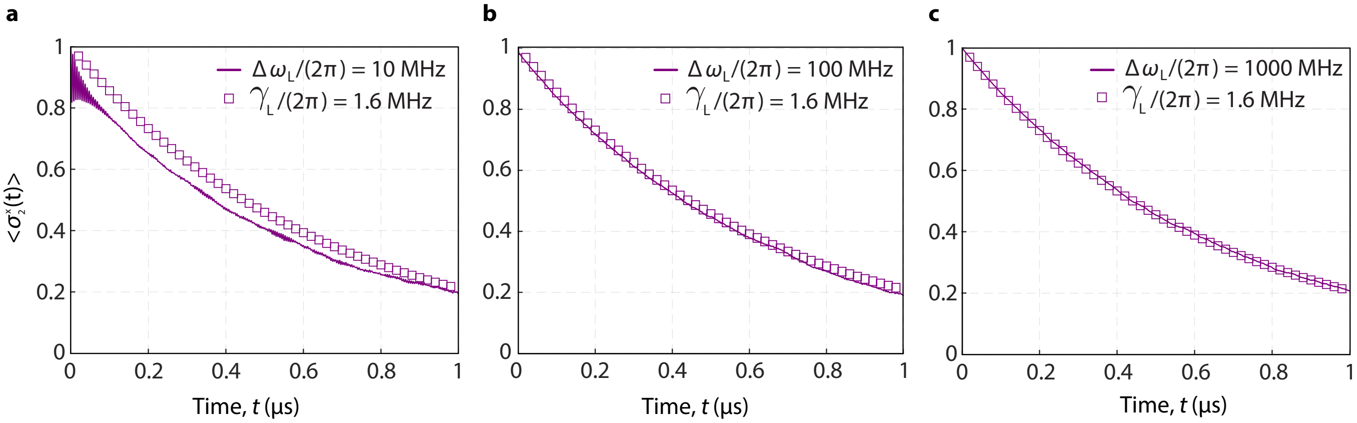

Supplementary Figure 7: Dephasing induced by Lorentizan noise. Dynamics of $\langle\sigma_2^x(t)\rangle$ as a function of Lorentzian noise power bandwidth $\Delta\omega_{\rm L}/2\pi = \{10,\,100,\,1000\}$ MHz. The noise amplitude A is decreased with increasing $\Delta\omega_{\rm L}$ so as to keep $\gamma_{\rm L}$ fixed at $\gamma_{\rm L}/2\pi = 1.6$ MHz. Open squares indicate a calculated exponential decay of $\langle\sigma_2^x(t)\rangle$ within the Markov approximation [see Eq. (41)] for $\gamma_{\rm L}/2\pi = 1.6$ MHz. As the Lorentzian noise correlation time $1/\Delta\omega_{\rm L}$ decreases (left to right), the system dynamics are increasingly more faithfully described within the Markov approximation [Eq. (41)].

The Hamiltonian [Eq. (33)] is non-demolition with respect to σ_2^z since $[\mathcal{H}_2, \sigma_2^z] = 0$, but leads to an effective decay of $\langle\sigma_2^x\rangle$ and $\langle\sigma_2^y\rangle$ components, which we derive next. The effective dephasing rate is extracted by studying the dynamics of these observables. Moving into a frame rotating at ω_2, we write the Heisenberg equations of motion for these operators as

$$\dot{\sigma}_2^x = -2\xi(t)\sigma_2^y, \tag{35}$$
$$\dot{\sigma}_2^y = +2\xi(t)\sigma_2^x. \tag{36}$$

The above coupled system is formally solved to obtain a single dynamical equation for $\sigma_2^x(t)$. Averaging this equation under noise realizations, we arrive at

$$\dot{\sigma}_2^x(t) = -4\int_0^t d\tau\ \langle\xi(t)\xi(t-\tau)\rangle\sigma_2^x(t-\tau). \tag{37}$$

Note that the noise autocorrelation appears in the above memory kernel; it is then possible to proceed via a Markovian approximation provided the noise correlations decay much faster than the relaxation dynamics of the system, which are themselves driven by the noise. We make this condition precise in a self-consistent way. Assuming Markovian approximation, we can drop the system's dependence on its past history via the memory kernel and extend the integral's upper limit to infinity, thus obtaining

$$\dot{\sigma}_2^x(t) = -4\sigma_2^x\int_0^\infty d\tau\ \langle\xi(\tau)\xi(0)\rangle. \tag{38}$$

The remaining integral is simply half the zero frequency power spectral density of the noise signal (ignoring the principle part that leads to a Lamb shift contribution, not dephasing), so that

$$\dot{\sigma}_2^x(t) = -2S_{\xi\xi}(0)\cdot\sigma_2^x. \tag{39}$$

Hence, the noise signal drives system decay at a rate $2S_{\xi\xi}(0)$ within the Markovian approximation. For white noise, which is δ-correlated, correlations always decay faster than the induced decay. Using $S_{\xi\xi}(0)$ for white noise, the dephasing rate γ_ϕ is given by:

$$\gamma_\phi = 2A_{\rm W} \tag{40}$$

More interesting is the case of Lorentzian noise. Eq. (39) yields a decay rate $\gamma_{\rm L}$ for this case as well, so long as the Lorentzian noise 'appears' white, namely when the correlation time of the Lorentzian noise signal is much shorter than the time scale of the decay it induces, $1/\gamma_{\rm L}$. Since the Lorentzian noise correlation time is on the order of its inverse bandwidth $1/\Delta\omega_{\rm L}$, we require $1/\Delta\omega_{\rm L} \ll 1/\gamma_{\rm L}$, or $\Delta\omega_{\rm L} \gg \gamma_{\rm L}$. If this is the case, the dephasing rate is given by

$$\gamma_{\rm L} = 2A_{\rm L}\frac{\left(\frac{\Delta\omega_{\rm L}}{2}\right)^2}{\omega_{\rm L}^2 + \left(\frac{\Delta\omega_{\rm L}}{2}\right)^2}\ ,\ \Delta\omega_{\rm L} \gg \gamma_{\rm L}. \tag{41}$$

Clearly, the dephasing rate is reduced compared to the white noise value for the same noise amplitudes ($A_{\rm W} = A_{\rm L}$). This is due to the colored noise spectrum which is not in fact equal at all frequencies.

However, we caution that the above expression holds only when the Lorentzian noise bandwidth is much larger than the induced decay rate, $\Delta\omega_{\rm L} \gg \gamma_{\rm L}$. In the current experiment, this condition is not met and the Markovian approximation should not hold. To observe dynamics in this regime for the simple single qubit model, we numerically compute the noise-averaged dynamics of $\langle\sigma_2^x\rangle$ as governed by $\mathcal{H}_2$, over multiple realizations of the noise $\xi(t)$. By varying the bandwidth $\Delta\omega_{\rm L}$ of the Lorentzian noise, we are able to explore both Markovian and non-Markovian regimes. For each $\Delta\omega_{\rm L}$, the noise amplitude A is chosen such that the decay rate within the Markovian

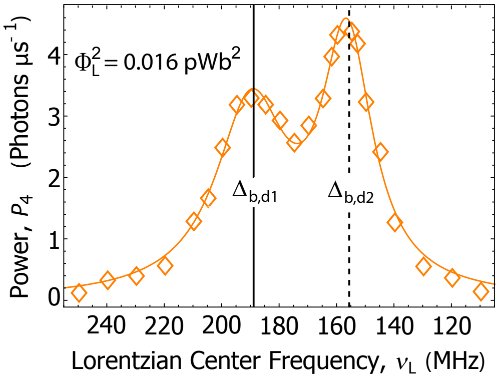

Supplementary Figure 8: Extracted power P_4 as a function of center frequency ν_L of Lorentzian noise spectrum. This measurement was performed at Lorentzian noise power $\Phi_L^2 = 0.016$ pWb^2 and coherent excitation. Orange solid line is a fit to a sum of two Lorentzians.

approximation has the fixed value $\gamma_L/2\pi = 1.6$ MHz. The dynamics of $\langle\sigma_2^x(t)\rangle$ (solid line in Supplementary Fig. 7) for $\Delta\omega_L/2\pi = \{10, 100, 1000\}$ MHz increasing from left to right is approximately an exponential decay. As $\Delta\omega_L$ becomes large in comparison to γ_L - that is, when the noise correlation time $1/\Delta\omega_L$ becomes increasingly short relative to the noise-induced system relaxation time $1/\gamma_L$ - the Lorentzian noise-induced dynamics approach those predicted within the Markovian approximation (open squares in Supplementary Fig. 7). Due to its non-Markovian effects Eq. (39) does not exactly describe the decay of coherence for the Lorentzian noise, however, it offers a meaningful estimation that is used when comparing the effects of the two types of noise.

Supplementary Note 12: Numerical Simulations with Lorentzian Noise

Lorentzian noise spectra employed in the discussed experiments have bandwidths $\Delta\omega_L$ that are on the order of, or slower, than the pertinent circuit decay rates. This implies that noise autocorrelation decay times surpass typical system relaxation timescales. As such, a noise environment structured in this way can give rise to non-Markovian dynamics of the system density matrix: the system's state at time t can be affected by its history over a time set by the autocorrelation time of the applied noise. Note that this is the case even if the noise signal itself is entirely independent of the system evolution, as in the present setup, where the noise is algorithmically generated. Integrating out the Lorentzian noise signal yields complex memory kernels that cannot be collapsed, unlike the case for Markovian dynamics. Our approach incorporates the Lorentzian noise environment as part of the system dynamics. In this way, we may still employ a Lindblad master equation for simulations of the system density matrix, at the cost of having to deal with a stochastic term describing the system's evolution.

To proceed, we add to the system Hamiltonian in Eq. (6) a modulation of the Q_2 energy splitting, given by the time series $\xi(t)$:

$$\mathcal{H}_\phi = \xi(t)\sigma_2^z \equiv \xi_0 \cos\left[\omega_L t + \phi(t)\right]\sigma_2^z \tag{42}$$

Here, $\phi(t)$ is a random variable describing phase noise, characterised by its statistical mean and variance:

$$\langle\phi\rangle = 0\ ,\ \langle\phi^2\rangle = \Delta\omega_L t \tag{43}$$

The variance being linear in time indicates that the phase undergoes diffusion, with the parameter $\Delta\omega_L$ characterizing the strength of this diffusion. The phase noise $\phi(t)$ is often referred to as Brownian noise or a Wiener process in other contexts and is the integral of Gaussian white noise. $\xi(t)$ has a Lorentzian power spectral density[14]

$$S_{\xi\xi}(\omega) = \xi_0^2 \frac{\frac{\Delta\omega_L}{2}}{(\omega-\omega_L)^2 + \left(\frac{\Delta\omega_L}{2}\right)^2} \tag{44}$$

with a constant integrated power proportional to ξ_0^2 (independent of the value of $\Delta\omega_L$). Note that $S_{\xi\xi}(\omega)$ is written for $\omega > 0$; a symmetrical contribution exists for negative frequencies since $\xi(t)$ is a classical signal. Finally, note that taking $\Delta\omega_L \to 0$ formally yields a coherent modulation of the Q_2 energy splitting at frequency ω_L.

For small ξ_0 we solve the master equation, Eq. (9), with the addition of $\mathcal{H}_\phi$ to the system Hamiltonian. The cost of adding a stochastic term to the system evolution is that any physical quantity must be computed via an explicit averaging procedure. In the Markovian approxiation, an equivalent procedure is implicitly carried out when 'tracing out the bath'. For a given set of system parameters, we propagate the Master equation to long times to obtain an approximate steady state density matrix ρ_{ss}. Then, steady state correlation functions are computed starting with the system in ρ_{ss}. To obtain meaningful results, these computations are repeated over multiple realizations of $\xi(t)$; the relevant resonator and transmission line power spectra are then given respectively by:

$$S_2(\omega) = \frac{\gamma_b}{2}\int_{-\infty}^{\infty} d\tau e^{-i\omega\tau}\langle(\sigma_b^+(\tau) - \langle\sigma_b^+\rangle_{ss})(\sigma_b^-(0) - \langle\sigma_b^-\rangle_{ss})\rangle_\phi \tag{45}$$

$$S_4(\omega) = \kappa\int_{-\infty}^{\infty} d\tau e^{-i\omega\tau}\langle a^\dagger(\tau)a(0)\rangle_\phi \tag{46}$$

where $\langle\cdot\rangle_\phi$ indicates an ensemble average over multiple realizations of $\phi(t)$. The subtraction of steady state averages from the bright state correlation function serves to remove the Rayleigh scattered peak from the transmission spectrum.

For stationary problems, a variety of methods exist to compute the above power spectra directly in the frequency domain, foregoing the need for a Fourier transform. Such techniques do not apply here, following inclusion of the explicitly time-dependent Lorentzian noise

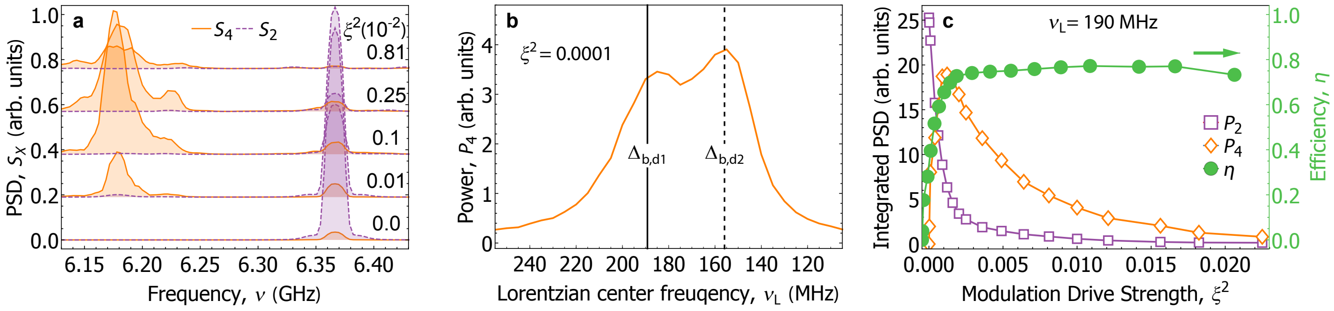

Supplementary Figure 9: Stochastically averaged master equation simulations. **a** Power spectral density calculated at the resonator $S_4(\omega)$ and waveguide $S_2(\omega)$ output for Lorentzian noise with a center frequency of $\nu_c = 190$ MHz and variable modulation drive strength ξ^2. **b** Calculated integrated power P_4 as a function of the Lorentzian center frequency ν_L at fixed modulation drive strength $\xi^2 = 0.0001$. **c** Integrated powers P_2 and P_4 and the transfer efficiency η as a function of Lorentzian noise modulation drive strength ξ^2. Power spectra at lowest noise power are matched with experiment to fix overall scaling factors, which are then used to scale all other spectra.

term. We find that the aperiodic, finite nature of computed correlation functions here leads to well known artifacts in their numerical Fourier transforms, namely a broad noise spectrum in the frequency domain due to spectral leakage. To suppress this noise, we apply a standard, total-power-preserving (Blackman) windowing function prior to performing the Fourier transform. This technique non-uniformly modifies the power at every frequency component, in different ways for each correlation function, restricting us to making only qualitative comparisons with experiment.

Simulated spectra $S_4(\omega)$ and $S_2(\omega)$ as a function of increasing Lorentzian noise power (Supplementary Fig. 9a), integrated power at the resonator P_4 as a function of Lorentzian center frequency ν_L (Supplementary Fig. 9b) and the integrated power at the resonator P_4, the open waveguide P_2 and the transport efficiency η as a function of Lorentzian noise power (Supplementary Fig. 9c) are all in good qualitative agreement with the experimental results (Figs. 3c, 4b and Supplementary Fig. 8).

Supplementary Note 13: Effective Qubit-Environment coupling

Engineered noise with Lorentzain PSD emulates coupling of Q_2 to a classical phononic mode at center frequency ω_L and spectral width $\Delta\omega_L$. We can estimate the effective qubit-environment coupling by decomposing the applied flux into a large static and a small fluctuating component $[\Phi(t) = \Phi_0 + \Delta\Phi(t)]$. Using Eq. (2) the qubit transition energy can be decomposed as

$$H_q/\hbar = \omega(t)\sigma_2^z = \omega_0\sigma_2^z + \sigma_2^z \left.\frac{d\omega}{d\Phi}\right|_{\Phi_0} \cdot \Delta\Phi(t). \qquad (47)$$

By assuming that a phononic environmental mode carries at most a single excitation its harmonic spectrum can be effectively substituted by that of a two-level system

$$H_q/\hbar \approx \omega_0\sigma_2^z + K\sigma_2^z \left[\sigma_{\rm ph}^x(t) + \sigma_{\rm ph}^{x\dagger}(t)\right], \qquad (48)$$

where $K = d\omega/d\Phi \cdot \Delta\Phi_0$ is an effective qubit-environment coupling constant and $(\sigma_{\rm ph}^x)$ is dimensionless Pauli operator with unit magnitude.

For experiments with Lorentzian noise we estimate the effective qubit-environment coupling constant (K) as a root-mean-square of Q_2 transition frequency fluctuation induced by applied structured noise.

Supplementary Note 14: Modulating the Transition Frequency of Q_2 with a Coherent Tone

In order to elucidate the mechanism of energy transport for Lorentzian noise applied to Q_2 we perform an additional measurement in which the Q_2 transition frequency is coherently modulated via the flux line while it is simultaneously coherently driven via the waveguide. This corresponds to the limiting case of very narrow noise power spectral density. We initially adjust the frequency of the coherent tone ν_c to be equal to the $|b\rangle$, $|d_1\rangle$ frequency difference $\Delta_{b,d1}$ as in the Lorentzian noise case.

Measurements of the power spectral density at the resonator $S_4(\omega)$ show, similarly to the Lorentzian noise case (see Fig. 3c), a pronounced resonance at $|d_1\rangle$ frequency composed of a broad part with linewidth of approx. 20 MHz and a strong narrow peak with the linewidth of approx. 500 kHz (see Supplementary Fig. 10a). The narrow peak is comparable in width with the environmental bright $|b\rangle$ state pure dephasing rate $\gamma_\phi^b/2\pi = 380$ kHz which indicates that it probably originates from the broadened and frequency shifted coherent microwave drive tone applied to the waveguide. When sweeping ν_c between 100 and 250 MHz and keeping the power of the modulation tone con-

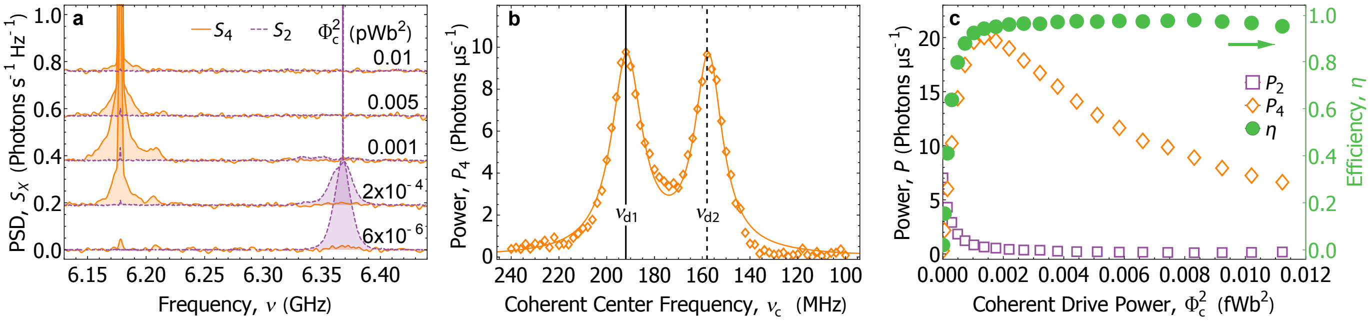

Supplementary Figure 10: Coherent environmental modulation of Q_2 transition frequency. **a** Measured power spectral densities of radiation extracted from the resonator $S_4(\omega)$ and re-emitted into the transmission line $S_2(\omega)$ for coherent modulation of Q_2 transition frequency with center frequency at $\nu_c = 190$ MHz and indicated powers Φ_c^2. **b** Integrated extracted power P_4 as a function of coherent modulation frequency ν_c at fixed modulation tone power $\Phi_c^2 = 54.6$ aWb^2. **c** Integrated powers P_2 and P_4 and the transfer efficiency η as a function of coherent modulation power Φ_c^2.

stant at $\Phi_c^2 = 54.6$ aWb^2, P_4 shows similar dependence as in the white noise case where the extracted power is maximized for $\nu_c = \Delta_{b,d1}$ or $\Delta_{b,d1}$ (Supplementary Fig. 10b). The linewidth of the resonances $\Delta\nu_{d1} = 14.1$ MHz and $\Delta\nu_{d2} = 15.7$ MHz correspond to the $|d_1\rangle$ and $|d_2\rangle$ state spectral widths. In the case of Lorentzian noise these were additionally broadened by the Lorentzian PSD width of $\Delta\nu_L = 10$ MHz, which resulted in $\Delta\nu_{d1,L} = 30.0$ MHz and $\Delta\nu_{d2,L} = 21.5$ MHz (see fit in Supplementary Fig. 8).

For the low frequency coherent modulation the integrated power extracted from the resonator P_4 for $\nu_c = \Delta_{b,d1}$ is almost twice as large as in the Lorentzian noise case (see Supplementary Fig. 10c and Fig. 4b), with approximately half of the power originating from the narrow peak at $|d_1\rangle$. The enhanced value of P_4 is in agreement with the model proposed in the main text. The integrated power of the radiation re-emitted into the waveguide P_2 is significantly smaller (0.7 Photons/μs) when P_4 reaches its maximum, as compared to white noise (Fig. 4a) or Lorentzian noise (Fig. 4b) case. As a result the internal transfer efficiency, as defined in the main text, reaches maximum values above 95%. Although not relevant for light-harvesting processes the depletion of the bright $|b\rangle$ state population is a result of coherent population trapping in the $|d_1\rangle$ state and electromagnetic induced transparency (EIT) of the bright $|b\rangle$ state. In our experiment EIT originates from the destructive interference between coherent excitation of the bright state $|b\rangle$ and strong coherent exchange between $|d_1\rangle$ and $|b\rangle$ state due to low frequency coherent modulation of Q_2 transition frequency[15,16].

Supplementary Note 15:
Excitation with incoherent microwave radiation

We engineer a broadband incoherent microwave signal by up-converting white noise (see Supplementary Note 5). In the up-conversion process the high-frequency LO2 tone is multiplied by a low-frequency signal generated with an AWG (see inset to Supplementary Fig. 11). In our experiment, low-frequency white noise with flat spectral density up to $\omega_c/2\pi = 450$ MHz and exponential cutoff with characteristic width $\Delta\omega/2\pi = 5.44$ MHz is up-converted using a coherent tone at $\omega_{LO2}/2\pi = 6.371$ GHz. The resulting high-frequency incoherent signal has a power spectral density with a constant amplitude that spans over a 900 MHz wide band centered at $\omega_B/2\pi = 6.371$ GHz as shown in Supplementary Fig. 11. The attenuated incoherent signal is applied to the sample at port 1, similar to the RF line in Supplementary Fig. 1.

Applying Lorentzian noise to the incoherently excited system with central frequency $\nu_L = 190$ MHz set at the $|b\rangle$-$|d_1\rangle$ frequency difference we observe no enhancement of the extracted power $S_4(\omega)$ at ν_{d1} relative to ν_{d2} (Supplementary Fig. 12a) as in the case of coherent excitation. Contrary to the coherent excitation where a multi-photon process is fixed in frequency by a coherent tone, for the incoherent excitation a multi-photon process occurs over a larger frequency range and therefore does not produce a pronounced peak at the $|b\rangle$-$|d_1\rangle$ frequency difference.

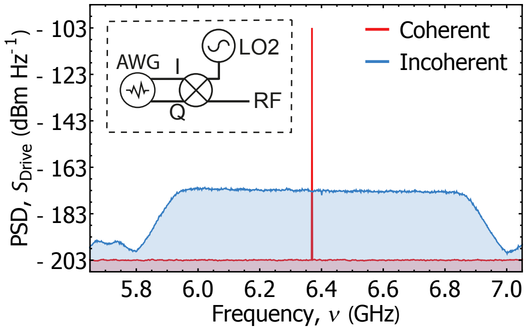

Supplementary Figure 11: Power spectral density of applied microwave signals. Comparison between incoherent and coherent microwave signal, measured with a spectrum analyzer. The inset shows a diagram of the up-conversion process.

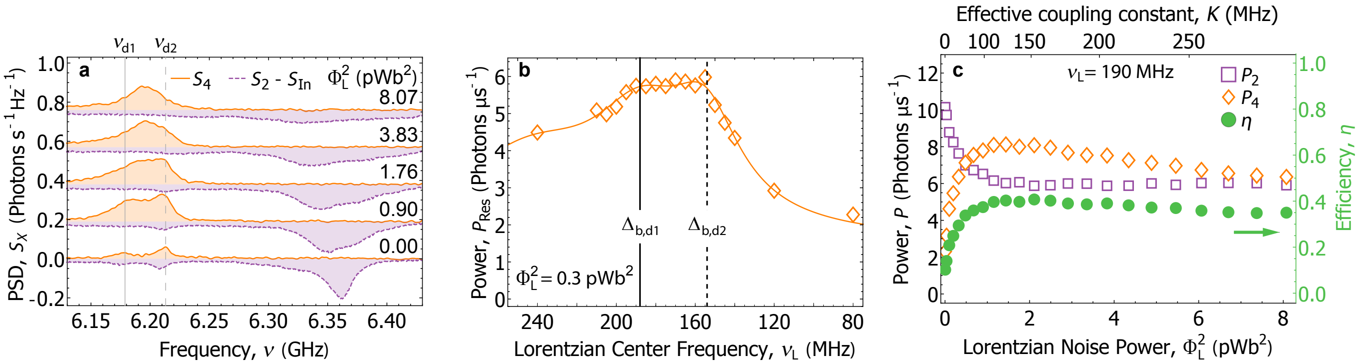

Supplementary Figure 12: Incoherently excited system subject to Lorentzian noise. **a** Power spectral density detected at the resonator $S_4(\omega)$ and at the waveguide $S_2(\omega)$ subtracted by separately measured incoherent radiation PSD as a function of Lorentzian noise power. **b** Integrated extracted power P_4 as a function of the center frequency ν_c of the Lorentzian noise at fixed noise power of $\Phi_L^2 = 0.3$ pWb^2. Solid orange line is a guide to the eye. **c** Integrated powers P_2 and P_4 and transfer efficiency η as a function of Lorentzian noise power Φ_L^2 or equivalently an effective qubit-environment coupling constant K. Lorentzian noise spectrum is centered at $\nu_c = 190$ MHz for **a** and **b**.

The Lorentzian noise increases the transferred power P_4 when resonant with the $|\mathrm{d}\rangle$ and $|\mathrm{d}_1\rangle$ or $|\mathrm{d}_2\rangle$ frequency difference (Supplementary Fig. 12b) similar to the coherent excitation case. The transfer efficiency η (Supplementary Fig. 12c) shows a non-monotonic behaviour as a function of applied Lorentzian noise power Φ_L^2 or effective coupling constant K with the maximum at $K/2\pi \approx 130$ MHz, similar to the coherent excitation case. However, the maximal efficiency $\eta_{\mathrm{L,inc.}}^{\max} = 41\%$ is considerably lower compared to the coherent excitation and Lorentzian noise. The reduced efficiency can be attributed to the absence of a resonant multi-photon process, which increases the efficiency of the coherently excited system. On the other hand, the maximal efficiency obtained with Lorentzian noise ($\eta_{\mathrm{L,inc.}}^{\max}$) is larger than the maximal efficiency obtained with white noise ($\eta_{\mathrm{W,inc.}}^{\max}$). This is in agreement with observations for the coherently excited qubit system. We conclude that the narrow Lorentzian noise spectrum enhances the excitation transport when resonant with the appropriate energy level mismatch for both coherently and incoherently excited qubit systems.

Supplementary References

[1] Barends, R. *et al.* Coherent josephson qubit suitable for scalable quantum integrated circuits. *Phys. Rev. Lett.* **111**, 080502 (2013).

[2] Koch, J. *et al.* Charge-insensitive qubit design derived from the Cooper pair box. *Phys. Rev. A* **76**, 042319 (2007).

[3] da Silva, M. P., Bozyigit, D., Wallraff, A. & Blais, A. Schemes for the observation of photon correlation functions in circuit QED with linear detectors. *Phys. Rev. A* **82**, 043804 (2010).

[4] Fink, J. M. *et al.* Quantum-to-classical transition in cavity quantum electrodynamics. *Phys. Rev. Lett.* **105**, 163601 (2010).

[5] Astafiev, O. *et al.* Resonance fluorescence of a single artificial atom. *Science* **327**, 840–843 (2010).

[6] Purcell, E. M. Spontaneous emission probabilties at radio frequencies. *Phys. Rev.* **69**, 681–681 (1946).

[7] Houck, A. A. *et al.* Controlling the spontaneous emission of a superconducting transmon qubit. *Phys. Rev. Lett.* **101**, 080502 (2008).

[8] Sete, E. A., Gambetta, J. M. & Korotkov, A. N. Purcell effect with microwave drive: suppression of qubit relaxation rate. *Phys. Rev. B* **89**, 104516 (2014).

[9] Carmichael, H. J. *Statistical Methods in Quantum Optics 1: Master Equations and Fokker-Planck Equations* (Springer, Berlin, 2002), 2 edn.

[10] Mollow, B. R. Power spectrum of light scattered by two-level systems. *Phys. Rev.* **188**, 1969–1975 (1969).

[11] Martinis, J. M., Nam, S., Aumentado, J., Lang, K. & C., U. Decoherence of a superconducting qubit due to bias noise. *Phys. Rev. B* **67**, 094510 (2003).

[12] Johansson, J. R., Nation, P. D. & Nori, F. QuTiP 2: A Python framework for the dynamics of open quantum systems. *Comput. Phys. Commun.* **184**, 1234–1240 (2013).

[13] Zhang, Y., Celardo, G. L., Borgonovi, F. & Kaplan, L. Opening-assisted coherent transport in the semiclassical regime. *Phys. Rev. E* **95**, 022122 (2017).

[14] Lax, M. Classical Noise. V. Noise in Self-Sustained Oscillators. *Phys. Rev.* **160**, 290–307 (1967).

[15] Abdumalikov, A. A. *et al.* Electromagnetically induced transparency on a single artificial atom. *Phys. Rev. Lett.* **104**, 193601 (2010).

[16] Novikov, S. *et al.* Raman coherence in a circuit quantum electrodynamics lambda system. *Nature Phys.* **12**, 75–79 (2016).
